# Wild-Type *α*-Synuclein and Variants Occur in Different Disordered Dimers and Pre-Fibrillar Conformations in Early Stage of Aggregation

**DOI:** 10.3389/fmolb.2022.910104

**Published:** 2022-06-28

**Authors:** Adrien Guzzo, Patrice Delarue, Ana Rojas, Adrien Nicolaï, Gia G. Maisuradze, Patrick Senet

**Affiliations:** ^1^ Laboratoire Interdisciplinaire Carnot de Bourgogne, UMR 6303 CNRS-Université de Bourgogne Franche-Comté, Dijon, France; ^2^ Schrödinger, Inc., New York, NY, United States; ^3^ Baker Laboratory of Chemistry and Chemical Biology, Cornell University, Ithaca, NY, United States

**Keywords:** α-synuclein, amyloid, Parkinson’s disease, molecular dynamics, dimers, CUTABI

## Abstract

*α*-Synuclein is a 140 amino-acid intrinsically disordered protein mainly found in the brain. Toxic *α*-synuclein aggregates are the molecular hallmarks of Parkinson’s disease. *In vitro* studies showed that *α*-synuclein aggregates in oligomeric structures of several 10th of monomers and into cylindrical structures (fibrils), comprising hundred to thousands of proteins, with polymorphic cross-*β*-sheet conformations. Oligomeric species, formed at the early stage of aggregation remain, however, poorly understood and are hypothezised to be the most toxic aggregates. Here, we studied the formation of wild-type (WT) and mutant (A30P, A53T, and E46K) dimers of *α*-synuclein using coarse-grained molecular dynamics. We identified two principal segments of the sequence with a higher propensity to aggregate in the early stage of dimerization: residues 36–55 and residues 66–95. The transient *α*-helices (residues 53–65 and 73–82) of *α*-synuclein monomers are destabilized by A53T and E46K mutations, which favors the formation of fibril native contacts in the N-terminal region, whereas the helix 53–65 prevents the propagation of fibril native contacts along the sequence for the WT in the early stages of dimerization. The present results indicate that dimers do not adopt the Greek key motif of the monomer fold in fibrils but form a majority of disordered aggregates and a minority (9–15%) of pre-fibrillar dimers both with intra-molecular and intermolecular *β*-sheets. The percentage of residues in parallel *β*-sheets is by increasing order monomer < disordered dimers < pre-fibrillar dimers. Native fibril contacts between the two monomers are present in the NAC domain for WT, A30P, and A53T and in the N-domain for A53T and E46K. Structural properties of pre-fibrillar dimers agree with rupture-force atomic force microscopy and single-molecule Förster resonance energy transfer available data. This suggests that the pre-fibrillar dimers might correspond to the smallest type B toxic oligomers. The probability density of the dimer gyration radius is multi-peaks with an average radius that is 10 Å larger than the one of the monomers for all proteins. The present results indicate that even the elementary *α*-synuclein aggregation step, the dimerization, is a complicated phenomenon that does not only involve the NAC region.

## 1 Introduction

*α*-Synuclein (*α*-syn) is a soluble 140 amino-acid intrinsically disordered protein (IDP) (Wright and Dyson, 1999) abundant in the brain (Jakes et al., 1994; Mollenhauer et al., 2008), the function of which remains unclear. Abnormal aggregation of *α*-syn is central to the onset of diseases, and its inhibition is an intensive research area (Teil et al., 2020). In synucleopathies (Soto, 2003; Chiti and Dobson, 2006; Stefanis, 2012; Chiti and Dobson, 2017; Tanudjojo et al., 2021), such as the Parkinson disease (PD), *α*-syn is found in high concentration, as filamentous aggregates, in intraneuronal inclusions (Lewy bodies) and in extracellular deposits (Lewy neurites) (Spillantini et al., 1997; Breydo et al., 2012; Lashuel, 2020; Trinkaus et al., 2021). In addition to sporadic PD, rare familial cases of PD with similar phenotypes are induced either by an overexpression of wild-type (WT) *α*-syn due to *α*-syn gene triplication or by pathogenic mutations in the *α*-syn gene corresponding to single amino-acid substitution (Polymeropoulos et al., 1997; Krüger et al., 1998; Zarranz et al., 2004; Fuchs et al., 2008; Appel-Cresswell et al., 2013; Pasanen et al., 2014; Petrucci et al., 2016).

The origin of aggregation of *α*-syn is unknown. The formation of *α*-syn amyloid shares many common properties with the toxic aggregation of other IDPs, as, for example, the aggregation of A*β* in Alzheimer’s disease (Cremades et al., 2017; Iadanza et al., 2018; Nguyen et al., 2021). When incubated in physiological conditions *in vitro*, *α*-syn aggregates spontaneously into fibrils with polymorphic cross-*β*-sheet conformations, in which a core of *β*-strands is aligned perpendicularly to the fibril axis, forming extended regular *β*-sheets with different arrangements (Tuttle et al., 2016; Guerrero-Ferreira et al., 2019; Guerrero-Ferreira et al., 2020). These different *in vitro* regular *β*-sheet structures are believed to mimic the *α*-syn aggregates in synucleopathies. Post-mortem X-ray diffraction of brains from patients with PD reveals patterns of *β*-sheet arrangements (Araki et al., 2019). The first *ex vivo* structures of *α*-syn fibrils from patients with multiple system atrophy have topology similar to the recombinant *in vitro* fibrils but with structural differences and occur with non-proteinaceous moieties (Schweighauser et al., 2020). In all known structures of fibrils, the monomer chain folds according to a three-layered L-shaped motif bearing resemblance to a Greek key motif (Guerrero-Ferreira et al., 2020). New toxic polymorphs were recently detected for which no structures are yet available (De Giorgi et al., 2020).

A recent study using solid-state nuclear magnetic resonance (NMR) of WT *α*-syn aggregation on phospholipid small unilamellar vesicles identified pre-fibrillar and early fibrillar species (Antonschmidt et al., 2021). NMR data suggest a pre-fibrillar segmental folding of the *α*-syn monomer in a three-layered L-shaped motif resembling one of the mature fibrils. The folding occurs with about the first half of the residues of the N-terminal region of the protein bounded to the phospholipid in an *α*-helical conformation (Antonschmidt et al., 2021). Another study of *α*-syn aggregation in solution identifies helix-rich intermediates in the transition of unstructured conformation *α*-syn into *β*-sheet-rich fibril formation during the growth of fibrils (elongation phase) (Ghosh et al., 2015). Interestingly, *α*-syn may also form ribbon aggregates (Bousset et al., 2013).

*α*-Syn fibrils are composed of thousands of *α*-syn monomers (Iadanza et al., 2018). Kinetic studies of *α*-syn aggregation showed that the onset of fibril formation is preceded by a long lag phase, indicating that the initial rate of aggregation is controlled by the nucleation of fibrils. It is hypothesized that pre-fibrillar aggregates of *α*-syn, *α*-syn protofibrils, i.e., oligomers composed of several dozen monomers occurring in the lag phase, might be the most toxic aggregates (Conway et al., 2000; Winner et al., 2011; Lázaro et al., 2014). These *α*-syn oligomers are transient intermediate species with heterogeneous structures that are consumed as formed fibrils (Cremades et al., 2017). Structural properties of *α*-syn oligomers were observed by different techniques, including single-molecule Förster resonance energy transfer (FRET), atomic force microscopy (AFM), small angle x-ray scattering (SAXS), small angle neutron scattering (SANS), transmission electron microscopy (TEM), and cryo-electron microscopy (cryo-EM) (Conway et al., 2000; Volles et al., 2001; Fredenburg et al., 2007; Giehm et al., 2011; Cremades et al., 2012; Lorenzen et al., 2014; Tosatto et al., 2015; Cremades et al., 2017; Bhak et al., 2018; Li et al., 2019; Zhou and Kurouski, 2020).

Single-molecule FRET provides information on the distance probability distribution between two fluorophores covalently linked to the *α*-synuclein molecule. The FRET study of pre-fibrillar oligomerization of *α*-syn (oligomers composed of 2–150 monomers) revealed two types of oligomers: type A with a low FRET efficiency (large internal distances) and type B with a high FRET efficiency (low internal distances) (Cremades et al., 2012; Horrocks et al., 2015; Tosatto et al., 2015). Type A occurred first in the kinetic of aggregation. Primary nucleation of *α*-syn may result in the creation of oligomers of type A from monomeric protein molecules that can grow through monomer addition, but they can also convert into type B oligomers (Cremades et al., 2012). The type B oligomers are toxic, more compact than the initially formed type A oligomers, and more proteinase-K-resistant than the type A and the monomer (Cremades et al., 2012). Unlike type B, type A oligomers dissociate in distilled water (Horrocks et al., 2015). Single-molecule FRET showed that missense mutations (A30P, A53T, and E46K) observed in familial forms of PD influence the kinetics of formation of oligomers and induce structural differences compared with the WT (Tosatto et al., 2015). The concentration of oligomeric species of variants (A30P, A53T, and E46K) in the lag phase of fibril formation is similar to the one of the WT, which indicates that the effect of the single amino acid substitutions on the oligomer structure might be more relevant for their neurotoxicity (Tosatto et al., 2015). Both type A and B oligomers are observed for mutants with a conversion rate from A to B higher for A53T and lower for A30P compared to the WT (Tosatto et al., 2015). Interestingly, for E46K mutants, it is not possible to separate clearly the two types of oligomeric species. Electron microscopy analysis of *α*-syn aggregation showed that the A30P mutant promotes the formation of pore-like protofibrils, whereas A53T promotes the formation of annular and tubular protofibrillar structures (Lashuel et al., 2002). The E46K variant generates annular oligomers similar to those observed for the A53T and A30P mutants (Fredenburg et al., 2007).

Spherical metastable oligomers, with no significant secondary structures, with a diameter of 100 Å, were identified by SANS (Bhak et al., 2018). Oligomers composed of about 30 monomers forming a compact core with a flexible shell were characterized by SAXS (Lorenzen et al., 2014). These ellipsoidal oligomers, of the order of 100 Å size, inhibit both the primary nucleation and the subsequent elongation steps of *α*-syn fibrils (Lorenzen et al., 2014). The structure of two main subgroups of small oligomers, composed of 18 and 29 monomers on average, with a pore-like shape was identified by cryo-EM (Chen et al., 2015). The *β*-sheet structure in these oligomeric species is predominantly antiparallel (in opposite to the parallel *β*-sheet structure of fibrils) and amounts to 35% of the residues. Interestingly, spherical oligomers observed by AFM-IR are also mainly composed of the anti-parallel *β*-sheet (Zhou and Kurouski, 2020). AFM studies of *α*-syn pre-fibrillar formation on different solid surfaces reported the observation of heterogenous oligomers with spherical, elongated, and annular shapes (Conway et al., 2000; Zhou and Kurouski, 2020). Chains of spherical oligomers were also observed (Conway et al., 2000). Oligomers are formed in the primary nucleation phase of fibril formation but can also be generated upon fibril disaggregation (Bousset et al., 2013; Cremades et al., 2017).

The observation of annular oligomers for the WT and mutants suggests that *α*-syn may form a pore-like *β*-barrel structure in membranes that disrupt the membrane integrity (Volles et al., 2001; Fredenburg et al., 2007; Laganowsky et al., 2012). Monomeric *α*-syn and fibrils did not show membrane permeabilization activity (Volles et al., 2001). Exposure of hydrophobic side chains of oligomers to solvent may facilitate membrane permeabilization and be a source of cellular dysfunction (Cremades et al., 2012). Excessive production of reactive oxygen species, which may lead to cell apoptosis, is induced by *α*-syn oligomers (Cremades et al., 2012). The selective binding between transmembrane protein LAG3 (lymphocyte-activation gene 3) and *α*-syn preformed fibril species is also suggested as a mechanism of neurotoxicity in which LAG3 facilitates the endocytosis of toxic oligomeric species and the neuron-to-neuron transmission of pathological *α*-synuclein aggregates (Mao et al., 2016). In addition, the observation of *α*-syn helical tetramers (Bartels et al., 2011; Wang et al., 2011; Dettmer et al., 2015; Cote et al., 2018; Lucas and Fernández, 2020) stabilized by hydrophobic interactions and salt bridges between the monomers (Cote et al., 2018) suggests another mechanism of toxicity related to the equilibrium between the tetrameric form and other (*β*-sheet) oligomeric species (Bartels et al., 2011). Replica exchange molecular dynamics (MD) biased by NMR data showed that helix-rich and *β*-strand-rich trimers and tetramers are stable and may represent a minor population of *α*-syn in solution (Gurry et al., 2013).

Although oligomers composed of dozens of *α*-syn monomers are identified as toxic (type B oligomers), the role of dimers in the neurotoxicity of *α*-syn cannot be discarded. It has been hypothesized that the critical rate-limiting step in the primary nucleation is the oxidative formation and accumulation of a dityrosine cross-linked dimer (Krishnan et al., 2003). The peak of accumulation of dimers coincides with the rapid onset of fibrillation for the WT and mutant (A53T and A30P) proteins. Dimer formation is accelerated for the A30P and A53T variants (Krishnan et al., 2003). It is worth noting that dimers have a detectable membrane permeabilization activity (Fredenburg et al., 2007). As dimerization is the most initial step in self-assembling of monomers, dimers may play an important role in the different pathways of aggregation leading to type B oligomers. *α*-Syn dimers are systematically observed in the lag phase of *α*-syn aggregation and were characterized by FRET (Cremades et al., 2012; Horrocks et al., 2015; Tosatto et al., 2015), fluorescence (Krishnan et al., 2003; Fredenburg et al., 2007; Lv et al., 2015), AFM (Yu et al., 2008; Zhang et al., 2018), and circular dichroism (Roostaee et al., 2013).

Dimerization between fluorophore-free WT and mutant (A30P, A53T, and E46K) *α*-syn monomers on a substrate and fluorophore-labeled monomers in solution was analyzed by internal reflection fluorescence microscopy (Lv et al., 2015). Two types of dimers were identified which differ by an order of magnitude in their lifetimes. For the WT, E46K, and A53T, the less stable (type 1) and most stable (type 2) dimers have a lifetime of the order of about 200–300 ms and about 3s, respectively (Lv et al., 2015). The variant A30P is significantly different with lifetimes of the order of 700 ms (type 1) and 5 s (type 2), suggesting a more stable dimeric structure (Lv et al., 2015). Type 2 dimers were also observed in single-molecule AFM spectroscopy findings of WT *α*-syn (Yu et al., 2008). The lifetimes (types 1 and 2) of the mutants are systematically longer than the one of the WT, which indicates that the missense mutations seem to increase the stability of the dimer aggregate. Most dimeric structures are of type 1, but A30P and A53T single amino-acid substitutions increase significantly the type 2 population. The type 2 dimer might be related to the B-type toxic oligomer (Tosatto et al., 2015). The *α*-syn dimers have a low FRET efficiency but might be a mixture of types A and B, which cannot be resolved experimentally (Horrocks et al., 2015). The fraction of dimers formed in the lag phase of aggregation is by increasing order WT < A53T < A30P as measured by FRET. The A30P variant generates the largest population of B oligomers at the end of the lag phase of fibrillation (Tosatto et al., 2015). These data (Lv et al., 2015; Tosatto et al., 2015) taken together indicate a significant structural difference between A30P dimers compared to WT and A53T dimers.

Differences between WT and mutant dimers were also observed by single-molecule AFM force spectroscopy at low pH (promoting aggregation) (Krasnoslobodtsev et al., 2013). Mutiple segments’ interactions between the monomers are favored in A53T and E46K compared to the WT as reflected by a larger number of multiple rupture force events (Krasnoslobodtsev et al., 2013), whereas A30P favored single segment dimerization compared to the WT (Krasnoslobodtsev et al., 2013). *α*-Syn monomer and dimer structures and dynamics were also measured at neutral pH by high-speed AFM for proteins adsorbed on a solid surface immersed in an aqueous solution (Zhang et al., 2018). The monomer adopts mainly a compact and stable spherical structure, but one-tail and two-tail transient structures were also identified (Zhang et al., 2018). Conformational transitions between different conformations occurred on a second-to-minute time scale. Dimers formed by the association of two globular monomers (major population) or of one globular monomer and a one-tail monomer (minor population) were observed (Zhang et al., 2018). However, the *α*-syn structure and dynamics on a surface might be different from those of *α*-syn in solution, as the confinement of a polymer on a surface is known to modify the dynamics and stability of the polymer conformations. For example, an *α*-syn monomer adopts an *α*-helical structure on the negatively charged (phospholipid) membrane (Fusco et al., 2018).

No experimental technique has so far provided the atomistic description of dimer heterogeneity. Detailed structural information on the influence of the missense mutations on the fundamental dimerization step of *α*-synuclein is missing. The structural properties and the role of *α*-syn dimers in different parallel pathways of the formation of larger oligomers are still unclear. MD simulation is a complementary means to single-molecule experimental techniques that may give insights on the dimerization process. Previous simulations of *α*-synuclein dimers were limited to the ultra-simplified model (discrete MD (Zhang et al., 2018) or small fragments of *α*-synuclein (Yamauchi and Okumura, 2021), which does not take into account properly the dynamics of the polymer or biased the initial conditions of the simulations by docking of the monomer native structure adopted by *α*-synuclein on a phospholipid membrane (Sahu et al., 2015). Here, we are going a step further by using unbiased replica exchange MD simulations of two *α*-synuclein molecules in an implicit solvent by using a physics-based coarse-grained UNited-RESidue (UNRES) force field (Maisuradze et al., 2010; Liwo et al., 2019) on a time scale of 29.7 milliseconds (72 replicas of 412 *μ*s each for each variant studied), which is three orders of magnitude larger than typical all-atom MD simulations (Khalili et al., 2005). The force field was calibrated to reproduce the structure and thermodynamics of small model proteins and applied with success to simulate protein folding (Maisuradze et al., 2010; Zhou et al., 2014; Sieradzan et al., 2021), large-scale conformational dynamics (Gołaś et al., 2012), A*β*-amyloids (Rojas et al., 2017), and the effect of A*β*-fibrils on the aggregation of tau protein (Rojas et al., 2018). In the present MD simulations, most of the *α*-syn molecules do not aggregate and remain thus in a monomeric conformation. These monomers for the WT, A30P, A53T, and E46K were recently described elsewhere (Guzzo et al., 2021). Here, we describe the structure and populations of the different dimeric states found in the MD simulations for the WT and the same mutants and compare those to data extracted from experiments.

## 2 Materials and Methods

All structures of *α*-syn (WT and A30P, A53T, and E46K variants) were extracted from replica exchange MD trajectories generated with the coarse-grained UNRES force field (Maisuradze et al., 2010; Liwo et al., 2019). In the UNRES force field, a polypeptide chain is represented as a sequence of *C* ^*α*^ atoms with united peptide groups located halfway of the virtual *C* ^*α*^–*C* ^*α*^ bonds and united side chains (SC) attached to the *C* ^*α*^ atoms. The SC–SC interaction potentials implicitly include the contribution from solvation (Liwo et al., 2001; Maisuradze et al., 2010). Descriptions of the UNRES force field and its parameterization are available in the reference (Liwo et al., 2019) and at http://www.unres.pl.

A total of 72 trajectories were computed for *each* protein: 32 trajectories at 300 K and 8 trajectories at each of the following temperatures: 310 K, 323 K, 337 K, 353 K, and 370 K, using replica exchange MD as described in Guzzo et al. (2021). Each trajectory was started with 2 fully unfolded monomers separated by a distance of 25 Å. The integration time step in UNRES is 4.9 fs corresponding to an effective actual time step of about 4.9 ps (Khalili et al., 2005). The convergence of each trajectory was monitored by computing the probability density of contacts of each residue, resulting in 30 million steps (effective time scale of 147 microseconds) of consolidated data out of 84 million steps (effective time scale of 412 microseconds) of simulation for *each* trajectory. The Cartesian coordinates of *C* ^*α*^ and SC beads were saved for every 1,000 integration steps. Only structures at 300 and 310 K (40 trajectories) are reported here as they are close to the physiological temperature.

Since the simulations are performed on two monomers, both isolated non-interacting monomer conformations and aggregated monomers were observed in the converged MD trajectories. A dimeric conformation was defined as two monomers with more than 10 pairs of residues with at least one intermolecular distance between their *C* ^*α*^ atoms smaller than 5 Å. This cut-off value was chosen because the average distance between two residues in an intermolecular *β*-sheet of an *α*-syn fibril is 4.8 Å (PDB ID: 2n0a). With this dimer definition, the fraction of dimers out of all the conformers simulated at 300 and 310 K is 31% for the WT, 23% for A30P, 29% for A53T, and 35% for E46K, respectively. The formation of dimers is increased for the E46K variant and is reduced for A30P (significantly) and A53T (weakly) mutants. The dimeric state of each protein is finally described here by about 300,000 structures extracted every 1,000 integration steps from the converged part of the replica exchange MD trajectories at 300 and 310 K, representing a sampling on an effective time scale of 1.47 milliseconds.

Analysis of the secondary structures of *α*-synuclein conformations was performed directly from the *C* ^*α*^ coordinates with the CUTABI (CUrvature and Torsion based of Alpha-helix and Beta-sheet Identification) algorithm recently developed in our group (Guzzo et al., 2021). CUTABI is 10–30 times faster than the commonly used DSSP algorithm (Dictionary of Secondary Structure of Proteins) (Kabsch and Sander, 1983; Touw et al., 2015) because it avoids the construction of an all-atom description of the protein backbone from the coarse-grained UNRES structure, as required to apply DSSP. In CUTABI, the minimal size of a helix is set to 4 residues. Helices with less than 3 residues, such as short 3_10_ helices, are thus not counted. The minimal size of a *β*-strand is set to 2 residues, i.e., a *β*-sheet cannot be smaller than 4 residues. The *β*-strands of 1 residue forming *β*-bridges are thus not considered. A detailed description of the MD trajectories and of the CUTABI algorithm can be found in our previous work on monomeric state (Guzzo et al., 2021).

**FIGURE 1:**
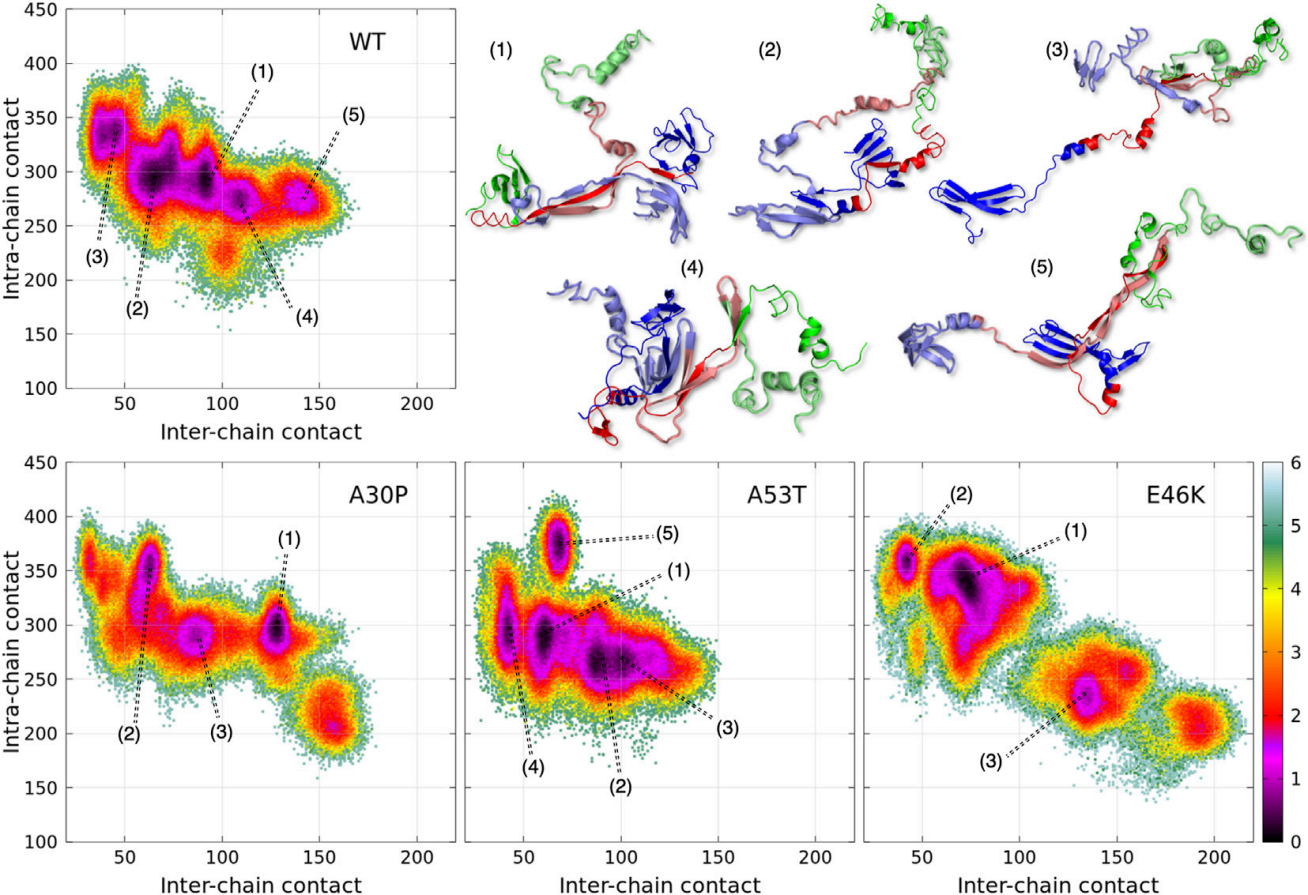
Color maps of - $\ln\left[\frac{P\left(n_{inter},n_{intra}\right)}{P_{max}}\right]$ computed from the 2D probability density function *P* of the number of inter-chain (*n*_*inter*_) and intra-chain (*n*_*intra*_) contacts between the residues of *α*-syn for the complete ensemble of dimers found in molecular dynamics trajectories at 300 and 310 K of the WT and variants. In each map, *P*_max_ is the maximum value of the probability of the map. The local minima within one unit from the global minimum of each map are numbered. Examples of 3D structures associated with each local minimum of the WT are represented with the N-terminus in blue, NAC in red, and the C-term in green. The light and dark colors differentiate the structure of the two monomers within the dimers.

**FIGURE 2:**
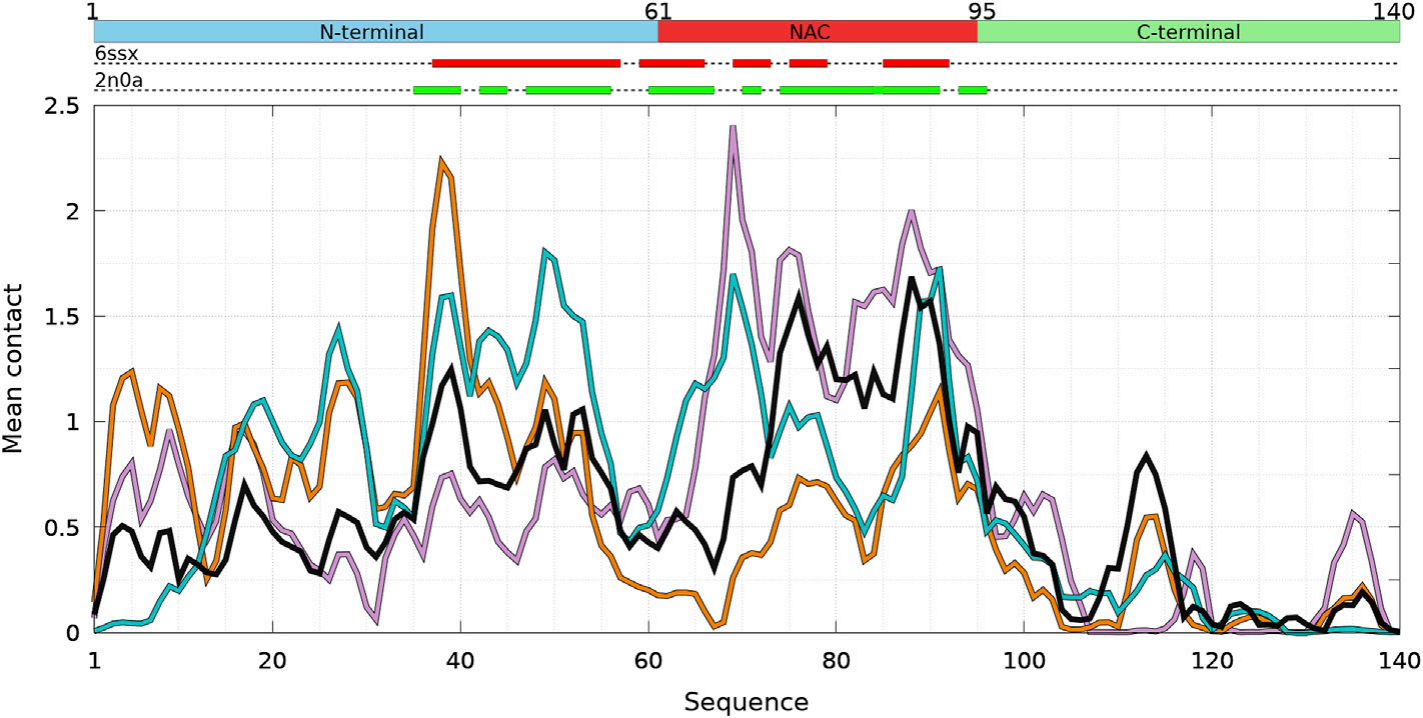
Mean intermolecular contact of a residue of *α*-syn WT and mutants for the complete ensemble of dimers found in molecular dynamics trajectories at 300 and 310 K. The color code is black (WT), violet (A30P), orange (A53T), and turquoise (E46K). The upper bar represents the N-terminal (blue), NAC (red), and C-terminal (green) regions of the sequence. The red and green rectangles on the upper dotted bars describe the locations of the intermolecular *β*-sheets identified by CUTABI in the experimental structures of *α*-syn fibrils with the PDB IDs 6ssx and 2n0a, respectively.

**FIGURE 3:**
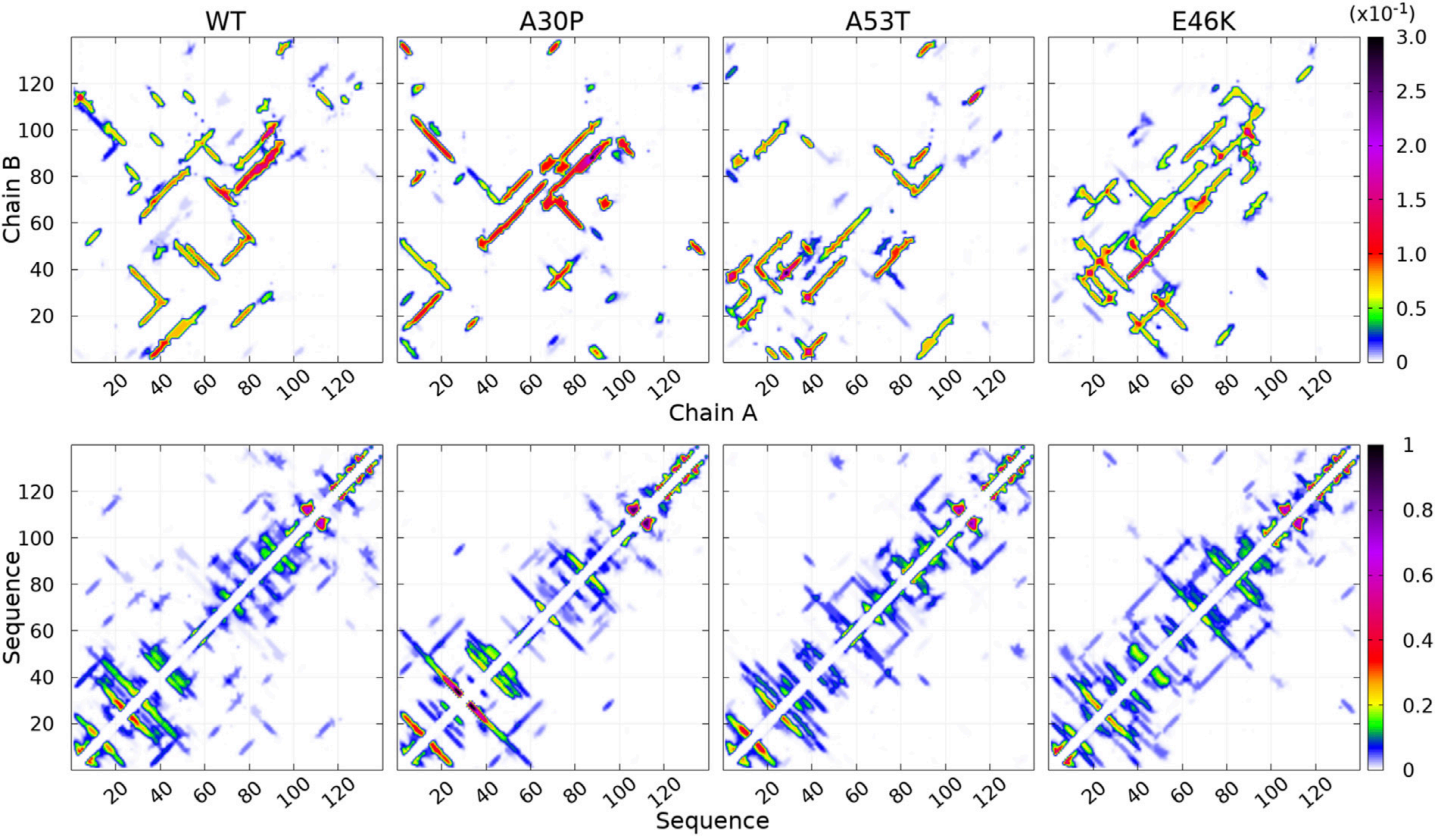
Contact maps computed for the complete ensemble of *α*-syn dimers found in molecular dynamics simulations for the WT and the mutants. The upper panels show intermolecular contacts between chains A and B of the dimers, and the lower panels show intra-molecular contacts within monomers A + B. The color bars indicate the probability. Note the different scales for intra- and intermolecular contact maps.

The statistics of contacts between amino acids (Figure 1, Figure 2, Figure 3) were computed by defining a contact as a pair of 2 *C* ^*α*^ atoms belonging to different residues at a distance smaller than 6 Å. By definition, the *C* ^*α*^ atom of a residue may form several contacts. The mean contact of a residue (Figure 2) is computed as the average of all *intermolecular* contacts made by this residue in all snapshots of all trajectories at 300 and 310 K.

## 3 Results

### 3.1 Propensity of *α*-Syn Dimerization

A dimensionless (effective) free-energy landscape of the *α*-syn dimers, - $\ln\left[\frac{P\left(n_{inter},n_{intra}\right)}{P_{max}}\right]$ , was computed from the two-dimensional probability density *P* of the number of inter-chain (*n*_*inter*_) and intra-chain (*n*_*intra*_) contacts between the residues for the WT and the mutants (Figure 1). *P*_max_ is the maximum value of the probability for each protein. For all proteins, *n*_*intra*_ varies between about 200 and 400. The minimum value of *n*_*inter*_ is 11 by definition of a dimer (see Material and Methods) and the maximum about 150 for the WT, A30P, and A53T and 200 for E46K. As for the monomer, the *α*-syn dimer has no unique native conformation but is represented by a large variety of dimer conformations. The structural diversity of the dimer conformations is shown in Figure 1 where one structure was randomly selected from each subpopulation of the minima of the WT effective free energy.

The numbered local minima correspond to different subpopulations of dimers with similar free energies (Table 1). These minima are separated by low barriers for the WT and A30P (violet to red regions between the minima in Figure 1). A larger barrier exists between the minima 1 and 5 of A53T and the minimum 3 and the minima 1 and 2 for E46K. It is worth noting that only the WT has a map without large barriers, whereas the maps of A30P, A53T, and E46K show small islands separated from the rest by large barriers. The inter-conversions between different subpopulations of dimer structures seem easier in the WT compared to the variants.

**TABLE 1:** Effective (dimensionless) free-energy difference (- $ln\left[\frac{P_{i}\left.\right)}{P_{1}}\right]$ ), where *P*_1_ and *P*_*i*_ are the probabilities of the minimum 1 and the of *i*th minima shown in Figure 1 for the WT and the variants.

Protein	Min 2	Min 3	Min 4	Min 5
WT	0.08	0.39	0.40	0.84
A30P	0.42	0.54	-	-
A53T	0.04	0.12	0.14	0.24
E46K	0.32	0.72	-	-

Figure 1 is muted on how the numerous intermolecular contacts are spread along the sequence. To answer this question, we first calculate the mean intermolecular contacts of residues along the sequence, as shown in Figure 2. For the WT, we observe seven representative peaks with maximum at A17, A27, Y39, and V49-A53 in the N-terminus; at A76 and I88-A90 in the NAC; and at L113 in the C-terminus. All these positions correspond to hydrophobic residues. In particular, the two largest peaks correspond to segments of three, A76-V77-A78, and four, I88-A89-A90-A91, hydrophobic residues, respectively. As mentioned previously, alanine plays a particular role in the *α*-syn dynamics (Cote et al., 2018). It is worth noting that the mean contact variable counts both intermolecular random coil contacts and intermolecular *β*-sheet contacts. However, in *in vitro* fibrils, as in dimers computed from MD, the intermolecular contacts are mainly from residues in *β*-sheets (see the next subsection). Therefore, we compare the mean contact curves with the location of intermolecular *β*-sheets computed by CUTABI in two different experimental *in vitro* structures of WT *α*-syn fibrils (PDB IDs: 6ssx and 2n0a) in Figure 2. The peaks at Y39 and V49-A53 in MD are within a region of *β*-sheets in both experimental WT fibril structures and the peaks at A76 and I88-A90. A more precise comparison between intermolecular *β*-sheets found in MD and these structures will be examined in Figures 6, 8.

As shown in Figure 2, it is remarkable to observe that a single mutation has huge effects on the relative weights of the different local regions having high aggregation propensity in the WT. The effects of a single amino-acid substitution are not limited to residues close to the mutation: the single amino-acid substitution has long-range effects on the mean contact. Compared to the WT, the main differences are as follows: 1) a larger propensity to aggregate in the N-terminal region for A53T and E46K and smaller for A30P, 2) a larger propensity to aggregate in the NAC region for A30P and lower for A53T, and 3) E46K has a propensity to aggregate larger than the WT all along the sequence excepted in region A76-I88 of the NAC. The largest peak of A30P is located at G69-V70-V71 corresponding to a succession of three hydrophobic residues. The highest propensity to aggregate is maximum at L38 for A53T and at V49 for E46K. The contribution of the C-term is modest and only significant close to L113 (except for A30P) and to Y136 (mainly for A30P).

How the different regions of the protein interact with each other is summarized by the intra-molecular and intermolecular contact maps in Figure 3 for the WT and the mutants. In such a map, the colored lines parallel to the diagonal indicate contacts between residues in parallel segments, whereas the colored lines perpendicular to the diagonal show contacts between residues in anti-parallel segments. The parallel lines are the most probable for the intermolecular contacts for all proteins. In the opposite, the perpendicular lines are the most probable and numerous for the intra-molecular contacts for the WT and mutants.

For the WT, the most probable intermolecular contacts are on the diagonal of the map in region 75–93 (NAC), which means that this region in one chain interacts with the corresponding region in the other chain. Such contacts are similar to those in fibrils. Using (A) and (B) for chains A and B, we find more precisely contacts between residues 75–85 (A) and 74–84 (B) and between 83 and 93 (A) and 83–93 (B). A similar region in the NAC has contact along a parallel to the diagonal, between residues 75–91 (A) and 84–100 (B). Other long regions of contacts shifted by several residues occur between residues 36–51 (A) and 2–17 (B), 32–52 (A) and 63–83 (B), 52–63 (A) and 88–99 (B) (parallel segments), and 52 and 66 (A) and 37–50 (B) (anti-parallel segments). There are also a large number of small contacts between parallel segments in different regions. An important characteristic of the WT map is the absence of intermolecular contacts along the diagonal in the region 50–65, which is favorable to the helical structure in the monomer (Guzzo et al., 2021). As mentioned, the contact map of intra-molecular contacts is characterized by a large number of anti-parallel segments in contacts for the WT. Using the numbers in the horizontal axis of the maps, the most probables are 2–23, 12–39, 14–47, and 33/36–50/51 (N-terminus) and 67–77 and 87–101 (NAC). The WT map shows a lot of weakly probable contacts in distant regions.

For A30P, the most probable intermolecular contact is along the diagonal or parallel to the diagonal between residues 38 and 95 (end of the N-terminus and NAC). Contacts strictly similar to those in fibrils are found between residues 80 and 95. Contacts between parallel segments close to the diagonal (shifted by several residues) are between residues 69–79 (A) and 73–83 (B), 38–67 (A) and 50–78 (B), and 17–25 (A). Probable anti-parallel intermolecular contacts are observed mainly between residues 3–23 (A) and 32–51 (B), and 8–25 (A) and 87–105 (B). Compared to the WT, the number of regions interacting with each other is less numerous. The intra-molecular contacts are mainly between anti-parallel segments in the N-terminus, as in the WT, with an extended region of high probability for segments 8–53 and 77–93.

For A53T, the most probable intermolecular contacts are in the N-terminal region [residues 1–60 (A)] with many contacts between shifted segments as, for example, 15–29 (A) and 41–55 (B), 26–35 (A) and 36–45 (B), and 37–55 (A) and 27–45 (B). Contrary to A30P, the WT, and E46K (discussed next), there are only short regions of intermolecular contacts parallel to the diagonal, namely, in the NAC region (85–92) and in the C-terminus (111–116). The intra-molecular contacts in the N-terminus are similar to those of the WT, whereas contacts in the NAC are similar to those of A30P.

The E46K mutant is characterized by a large region of high probability of aligned intermolecular contacts (on the diagonal) from the N-terminus to the NAC (regions 36–81 and 87–90) and a large region of parallel intermolecular contacts between shifted segments: between regions 15–95 (A) and 35–109 (B). Intra-molecular contacts between anti-parallel segments are found in regions 7–20, 12–38, and 72–94.

### 3.2 Heterogeneity of Secondary Structure Elements in *α*-Syn Dimers

**FIGURE 4:**
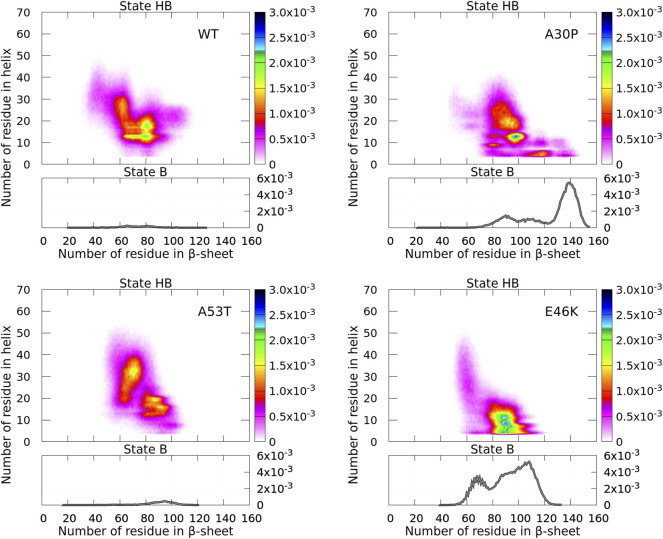
Probability density of the number of residues in *α*-helices and *β*-sheets for the WT and mutants of *α*-syn. The probability density of state B (no helix) is represented by a function (gray) (right vertical axis), and the probability density of state HB is represented by a two-dimensional map (right color bar).

For each protein, the algorithm CUTABI (Guzzo et al., 2021) was applied to the ensemble of *α*-syn dimers to compute the sum of residues in the *α*-helix (*α*) and in the *β*-sheet (*β*). Each dimer conformation thus has (*α*, *β*) coordinates. The resulting probability densities in the (*α*, *β*) space are represented in Figure 4. In these maps, only residues from the N-terminal and NAC regions were considered for the calculations as the C-terminal region does not contribute to secondary structure differences between the WT and mutants (Guzzo et al., 2021).

For the isolated monomers, a major observation was that the conformations of the monomers were divided into two distinct states for the N-terminal + NAC region: an ensemble of conformations with no residue in the helix (state B) and the rest of conformations (state HB). The highest probability of observing a conformation in state B was an order of magnitude larger than that of state HB (Guzzo et al., 2021). In Figure 4, states B are clearly visible for the monomers forming A30P and E46K dimers with a probability only twice larger than state HB. On the opposite, states B disappeared in the WT and A53T dimers where the probability to find a dimer with no helix is completely negligible. For A30P, the maximum number of residues in *β*-sheets is 155 and the highest probable number is 139, i.e., half of the residues of the dimer. A sub-state B is found at about 90 for A30P. For E46K, the maximum number of residues in state B is also large (133) with a peak at 108 and a second peak at 66.

**FIGURE 5:**
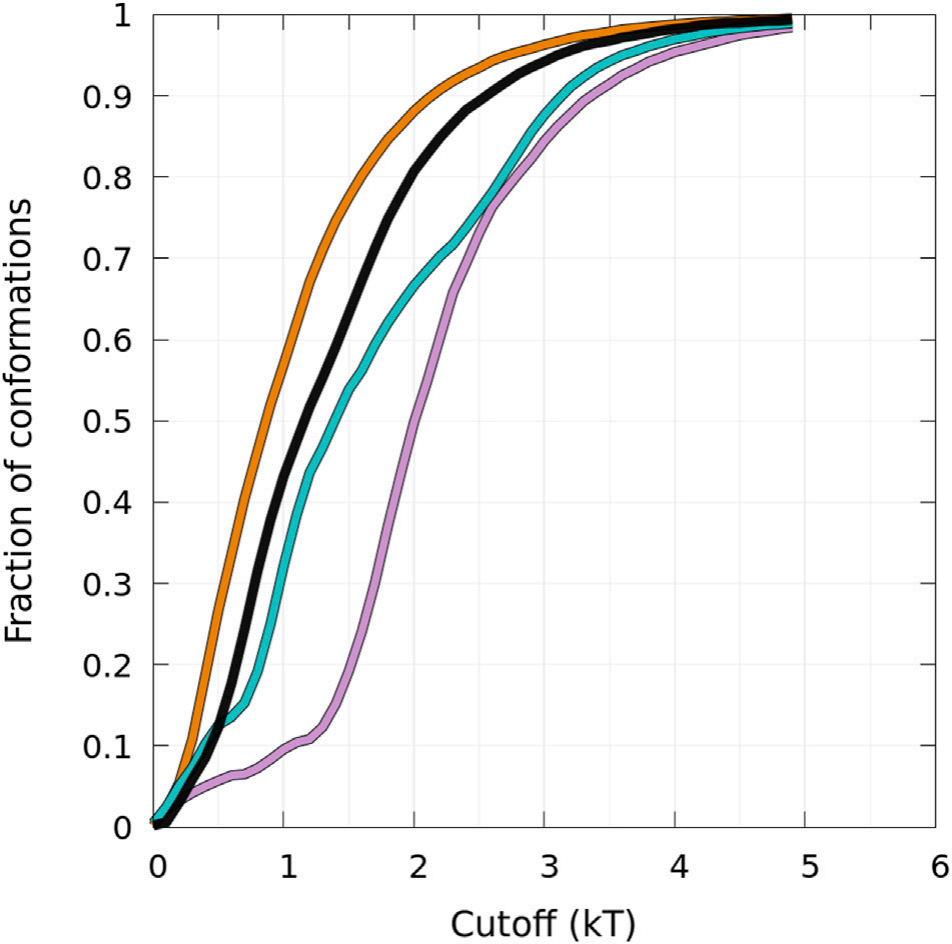
Cumulative fraction of the total number of conformations simulated as a function of an effective free-energy cut-off from the global minimum of the B state (see text) represented in Figure 4. The curves are for the WT (black), A30P (purple), E46K (turquoise), and A53T (orange).

States B and HB can be distinguished from the function presented in Figure 5 showing the fraction of conformations within an effective free-energy difference cut-off from the global minimum of state B for each protein (Guzzo et al., 2021). With *P*_max_, the maximum of probability at (0, *β*) (in the B state), and *P*, the probability at (*α*, *β*) (*α* ≥ 0, in the B or HB states), the effective free-energy cut-off is computed as $-\ln\left(\frac{P}{P_{max}}\right)$ in *kT* units, where *k* is the Boltzmann constant and *T* is the temperature. By definition, the *derivative of the curves* represented in Figure 5 represents the Density Of conformations or micro-States (DOS). For A30P and E46K, a change in slope (DOS) is observed at about 1.2 kT, corresponding to about 10 and 45% of the conformations, respectively. The change in slope at 1.2 kT points up the separation between main states, i.e., the onset of state HB, i.e., a state with a mixture of *α*-helices and *β*-sheets. The case of E46K is special. We observe in fact two changes in slope at about 0.8 and 1.2 kT corresponding to the major peak at 110 residues and the minor peak at 70 residues in the one-dimensional probability density of state B in Figure 4. The HB state of E46K (Figure 4) is also very different from the HB states of the other proteins: they are rare dimers with helical regions larger than about 15 residues. Conformations with a maximum number of residues in helix are a few on the time scale of the present simulations with a maximum number of residues in the helix of 59, 50, 63, and 61 for the WT, A30P, A53T, and E46K, respectively. As for the monomers, A53T is the structure with the largest number of residues in helix (Guzzo et al., 2021). It is worth noting that the free-energy map of the HB state of A53T is also the most diffuse. The global minima of the HB maps are (13,80), (13,98), (31,70), and (8,91) for the WT, A30P, A53T, and E46K, respectively. The positions of these minima reflect also the largest propensities to form helical segments and *β*-sheets for A53T and E46K, respectively.

**FIGURE 6:**
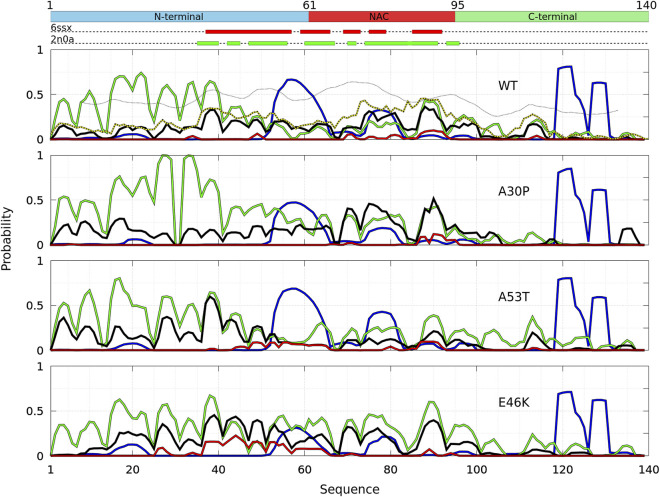
Analysis of the secondary structures for the complete ensemble of *α*-syn dimers found in molecular dynamics simulations for the WT and the mutants using the CUTABI algorithm (Guzzo et al., 2021). Probabilities for each residue to pertain to an *α*-helix (blue), to an intra-molecular *β*-sheet (green), to an intermolecular *β*-sheet (black), and to a native intermolecular *β*-sheet fibril type (red). The probability for each residue to form an intermolecular contact (Figure 2) is shown for the WT (black/yellow broken line) for comparison. The propensity to form a *β*-sheet calculated from the empirical scale of Chou and Fasman with the Protscale online tool (https://web.expasy.org/protscale/) using a window of 15 residues and a linear interpolation with a weight of 0.10 for the window edges is also represented (dotted line) for the WT. The red and green rectangles on the upper dotted bars describe the locations of the intermolecular *β*-sheets identified by CUTABI in the experimental structures of *α*-syn fibrils with the PDB IDs 6ssx and 2n0a, respectively.

The probability to find the different secondary structures along the amino-acid sequence of the WT and mutants of *α*-syn dimers was analyzed with CUTABI. As shown in Figure 6, helices are found in four main regions: two in the C-terminal region (residues 119–125 and 127–130), one in the NAC region (residues 75–82), and one overlapping the N-terminal and NAC regions (residues 53–65) for both the WT and mutants. There is no significant difference between the propensities of helices for the WT and mutants in the C-terminal region. These results for the helical propensity are similar to those found for the isolated monomers (Guzzo et al., 2021). The main differences are that the probability to form the helix 53–65 is twice larger in the dimer than in an isolated monomer for the WT and that the probability to form the 75–82 helix is about twice smaller in the dimer than in an isolated monomer for A30P and E46K.

The intra-molecular and intermolecular *β*-sheets were analyzed separately. The probability for a residue to pertain to an intra-molecular *β*-sheet has peaks at the same positions than for an isolated monomer. For the WT, the maximum of the peaks observed in Figure 6 is as follows (in brackets for an isolated monomer (Guzzo et al., 2021)): *P*_*F*4_ = 0.44 (0.57), *P*_*K*10_ = 0.53 (0.61), *P*_*A*17_ = 0.71 (0.87), *P*_*T*22_ = 0.74 (0.76), *P*_*E*28_ = 0.60 (0.65), *P*_*K*34_ = 0.64 (0.61), *P*_38_ = 0.65 (0.69), and *P*_*Y*39_ = 0.63 (0.69); *P*_*K*43_ = 0.36 (0.50), *P*_*V*49_ = 0.26 (0.4), *P*_53_ = 0.19, and *P*_*V*55_ = 0.13 (0.69); *P*_*V*63_ = 0.14 (0.64), *P*_*V*70_ = 0.15 (0.32), *P*_81_ = 0.18 (0.35), and *P*_*K*80_ = 0.17 (0.41); *P*_88_ = 0.43 and *P*_*A*90_ = 0.42 (0.58); and *P*_*V*95_ = 0.20 (0.35) and *P*_97_ = 0.27. These peaks are at the same locations for the mutants as they were in isolated monomers. These maxima are located at or close to valine residues, which is the most frequently found amino acid in *β*-sheets (Chou and Fasman, 1974). The empirical prediction of the dimensionless propensity of the formation of the *β*-sheet computed from the scale of Chou and Fasman (CF) (Chou and Fasman, 1974) is shown in Figure 6 for the WT. The CF simple algorithm, using only the primary sequence, predicts some peaks at similar locations than predicted by MD, namely, at 39, 52, 71, and 94. Of course, there are quantitative differences in the shape and relative heights predicted by CF compared to MD (they are not exactly the same quantities), but both methods identify qualitatively similar local regions of sequences which are important for *β*-sheets. It is worth noting that the CF algorithm predicts only a relatively small local change of the β-sheet propensity for the mutants, as the influence of a residue is limited to a few residues in the vicinity of its position.

Compared to the WT, the increase in probability of intra-molecular *β*-sheets in the region 26–35 for A30P and its decrease in the region 53–65 for A53T were also found in the isolated monomers (Guzzo et al., 2021). Compared to isolated monomers, the probability for a residue to pertain to an intra-molecular *β*-sheet decreases significantly from residues 43 to 100 for the WT, A30P, and A53T. Therefore, the intra-molecular *β*-sheets form mainly in the N-terminal region for these three proteins. For E46K, they are found equally in the N-terminal and NAC regions. Note that the CF algorithm underestimates the large propensity of formation of *β*-sheets in the N-terminal region.

The maxima of probability to form intermolecular *β*-sheets are located at the same positions than those of the probability to form intra-molecular *β*-sheets. The amplitude of these peaks is however different between the proteins. They are four major peaks for the WT and A30P: *P*_*Y*39,*WT*_ = 0.33, *P*_*T*75,*WT*_ = 0.31, *P*_*T*81,*WT*_ = 0.29, and *P*_*I*88,*WT*_ = 0.36, and *P*_*V*70,*A*30*p*_ = 0.40, *P*_*T*75,*A*30*p*_ = 0.46, *P*_*I*88,*A*30*p*_ = 0.40, and *P*_*A*90,*A*30*p*_ = 0.52, respectively. Except for the peak at Y39, the larger propensities to form intermolecular *β*-sheets are located in the NAC for the WT and A30P. For A53T, all the major peaks are in the N-terminal region: *P*_*F*4,*A*53*T*_ = 0.36, *P*_*E*28,*A*53*T*_ = 0.37, *P*_*L*38,*A*53*T*_ = 0.60, and *P*_*K*43,*WT*_ = 0.34. For the variant E46K, the propensity to form intermolecular *β*-sheets is mainly in the N-terminal region with peaks at *P*_*A*27,*E*46*K*_ = 0.35, *P*_*Y*39,*E*46*K*_ = 0.45, *P*_*T*44,*E*46*K*_ = 0.37, *P*_*V*49,*E*46*K*_ = 0.40, and *P*_*V*53,*E*46*K*_ = 0.35 but also in the NAC region with peaks at *P*_*V*71,*E*46*K*_ = 0.31 and *P*_*A*90,*E*46*K*_ = 0.40. A single amino-acid substitution modifies significantly the most probable regions of the formation of intermolecular *β*-sheets: highest in the NAC for the WT and A30P, in the N-terminal region for A53T, and in both the N-terminal region and in the NAC for E46K. These results compare well with the mean intermolecular contact profiles (Figure 2). As shown in Figure 6 for the WT, the mean contact curve follows quite well the probability of intermolecular contact (except for the 80–85 region). Finally, the probability to form intermolecular *β*-sheets between the same residues in both monomers (diagonal in the contact maps in Figure 3), named native fibril-like contacts (Nfcs), is also shown for comparison in Figure 6. For all proteins, the probability of Nfcs is low. The sub-ensemble of dimers showing these contacts is analyzed and discussed next.

### 3.3 Dimers With Fibril Native Contacts (Dfncs)

Contact maps of the WT and mutants show contacts along their diagonal as in the experimental structures of fibrils (PDB IDs: 6ssx and 2n0a). To identify conformations belonging to the diagonal of the contact maps, we extracted from the dimer statistics a sub-ensemble of dimers with Nfcs. We define a dimer with fibril-like native contacts (Dfncs) as a dimer conformation that has at least 5 consecutive intermolecular contacts made between residues with the same indices at a contact distance less than 5Å. This value was chosen as the distances between native contacts in fibrils are 4.9Å and 4.7Å in the measured structures with PDB IDs 6ssx and 2n0a. We may consider the Dfncs ensemble as conformations that are the most probable pre-formed fibrils in the present work.

**FIGURE 7:**
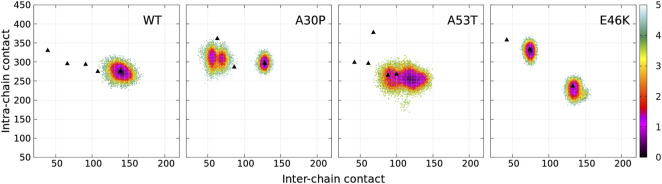
Color maps of - $\ln\left[\frac{P\left(n_{inter},n_{intra}\right)}{P_{max}}\right]$ computed from the 2D probability density function *P* of the number of inter-chain (*n*_*inter*_) and intra-chain (*n*_*intra*_) contacts between the residues of *α*-syn for the sub-ensemble of dimers with fibril native contacts found in molecular dynamics trajectories at 300 and 310 K of the WT and variants. In each map, *P*_max_ is the maximum value of the probability of the map. The local minima numbered in Figure 1 are shown by black triangles.

The dimensionless (effective) free-energy landscape of the Dfncs was computed from the two-dimensional probability density *P* of the number of inter-chain (*n*_*inter*_) and intra-chain (*n*_*intra*_) contacts between the residues for the WT and the mutants (Figure 7). For the WT and A53T, the Dfncs is located in one basin, whereas for A30P and E46K, two distinct clusters are visible. Some of the fibril clusters are located at the local minima of the global free-energy landscape (Figure 1), except for A53T. For A30P and E46K, one of the two clusters of Dfncs occurs at the global minimum of the global free-energy landscape (Figure 1). This indicates that Dfncs are more probable to be formed for these two mutants. In fact, the sub-ensemble of Dfncs represents 14.04% and 15.73% for A30P and E46K, respectively. For the WT and A53T, the population of Dfncs is only 8.33 and 10.65%, respectively. It is worth noting that these numbers indicate that the majority of dimers are disordered aggregates at least on the effective time scale (millisecond) of the present simulations.

**FIGURE 8:**
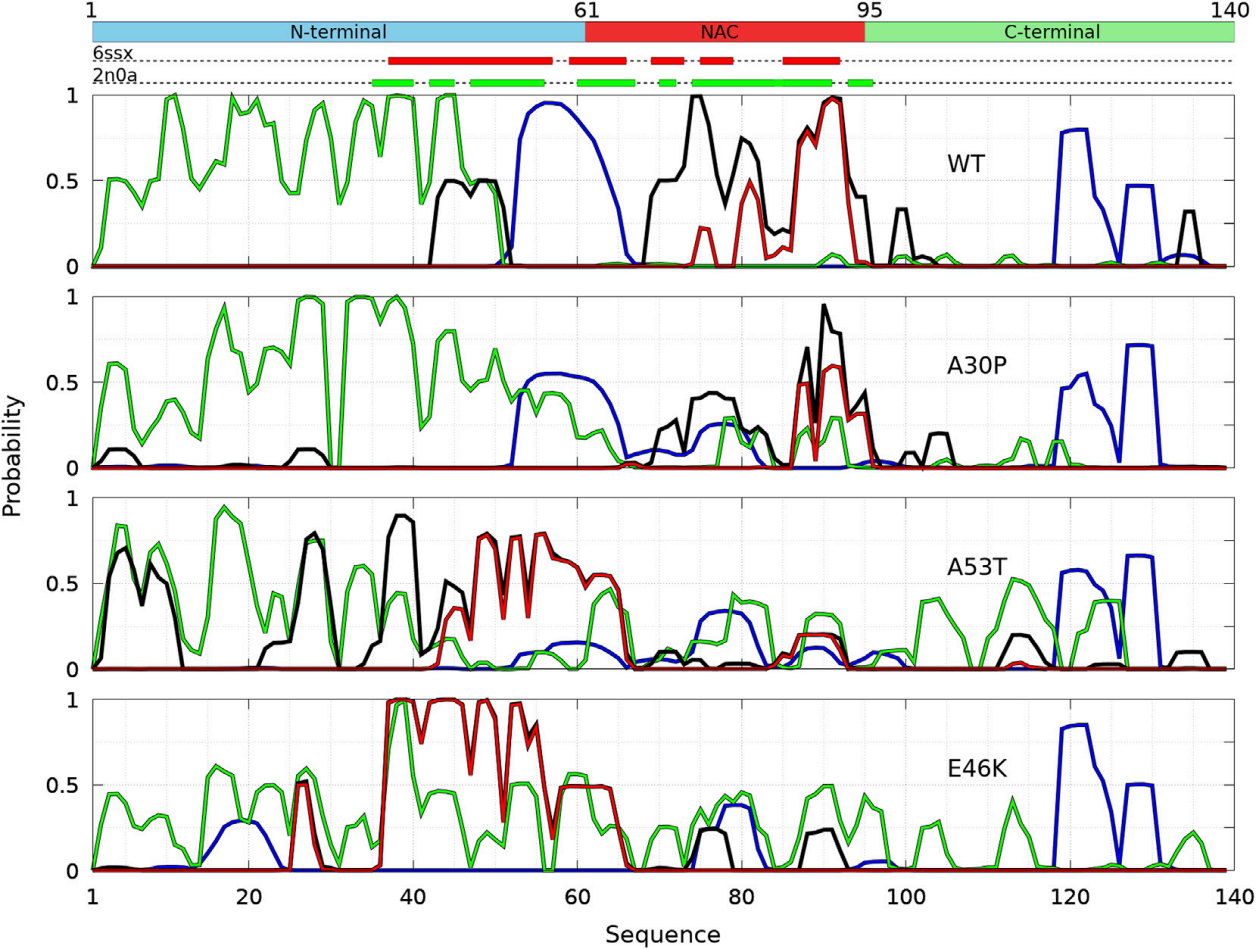
Analysis of the secondary structures in the sub-ensemble of *α*-syn dimers with native fibril-type contacts found in molecular dynamics simulations for the WT and the mutants at 300 and 310 K. Probabilities for each residue to pertain to an *α*-helix (blue), to an intra-molecular *β*-sheet (green), to an intermolecular *β*-sheet (black), and to a native intermolecular *β*-sheet fibril type (red).

The calculation of the probability to find the different secondary structure elements along the amino-acid sequence of the WT and mutants of *α*-syn dimers was repeated for the sub-ensemble of Dfncs using CUTABI (Figure 8). Compared to the ensemble of dimers, the helical regions of Dfncs show significant differences for the WT and A53T: the region 53–65 can only form a helix in the WT, preventing the dimerization of this region, whereas this helical region disappears in A53T and the helical region 75–82 disappears in the WT.

The peaks of probability for a residue to pertain in an intra-molecular *β*-sheet occur at the same positions in the Dfncs sub-ensemble and in the ensemble of the dimers for all proteins (see Figure 6). For the WT, the propensity of intra-molecular *β*-sheets is strongly increased in the N-terminal region of Dfncs with the peaks with a probability near 1 meaning residues pertaining to this type of secondary structure in all Dfncs conformations. On the contrary, all residues above position 50 have a totally negligible probability to form an intra-molecular *β*-sheet in Dfncs. Unlike the ensemble of WT dimers for which intermolecular *β*-sheets can be found nearly everywhere along the sequence (Figure 6), in the Dfncs sub-ensemble, they are found only for residues 43–51 (N-terminal region), 69–95 (NAC region), and 99–100 and 134–135 (C-terminal region). We observe a propensity to form Nfcs, as in fibrils, only for specific regions in the NAC: residues 75–76, 80–82, 85, and 87–93. For the segment 87–93, the superposition of the black and red curves in Figure 8 means that only Nfcs are formed there. Considering the limited time scale of the present simulations, these residues are probably nucleation centers of pre-fibril-like dimers in the WT.

For A30P, the probability to form intra-molecular *β*-sheets is similar in Dfncs and in the full ensemble of dimers (Figure 6) but with an increase in the region 36–58. Contrary to the ensemble of A30P dimers, for which intermolecular *β*-sheets can be found nearly everywhere between residues 3–104 along the sequence (Figure 6), in Dfncs, they are found in the NAC for residues 70–83 and 87–96 as well as with smaller probabilities in the C-terminal region at 103–105 and in the N-terminal region at 18–20 and 26–28. The propensity to form Nfcs occurs only in the NAC at 87–88 and 90–95, which are probably the nucleation centers for this type of structure for this variant. Clearly, the WT and A30P share the same region of formation of Nfcs in the NAC.

For A53T, the propensity for a residue to pertain to an intra-molecular *β*-sheet increases in the NAC and C-terminal regions in Dfncs compared to the complete ensemble of dimers (Figure 6). The formation of intermolecular *β*-sheets occurs all along the sequence: residues 3–11, 22–30, and 35–60 (N-terminal region); 61–65, 71, and 87–92 (NAC region), and 112–116 (C-terminal region). A53T differs thus from the WT and A30P by a large number of regions of intermolecular *β*-sheets, including the N-terminal region. In addition, the probability of segments 44–65 and 87–92 in the NAC to form Nfcs is identical to the probability of intermolecular *β*-sheets, meaning that these residues form only Nfcs in the Dfncs sub-ensemble and are probably nucleation centers for A53T pre-fibril-like dimers.

For E46K, the probability of intra-molecular *β*-sheets is similar between Dfncs and the ensemble of dimers. The exception is the peak at residues 38 and 39 for which the probability is 1 as well as the probability for these residues to form Nfcs, meaning that these residues are involved in both Nfc and intra-molecular *β*-sheets. As for the other proteins, the presence of intermolecular *β*-sheets is limited to specific regions in contrast to the ensemble of dimers where they were found along all the sequence (Figure 6). In addition, nearly all intermolecular *β*-sheets are Nfcs (the black and red curves in Figure 8 overlap), namely, at 26–28 and 37–60 (N-terminal region) and 61–65, 75–78, and 88–92 (NAC).

**FIGURE 9:**
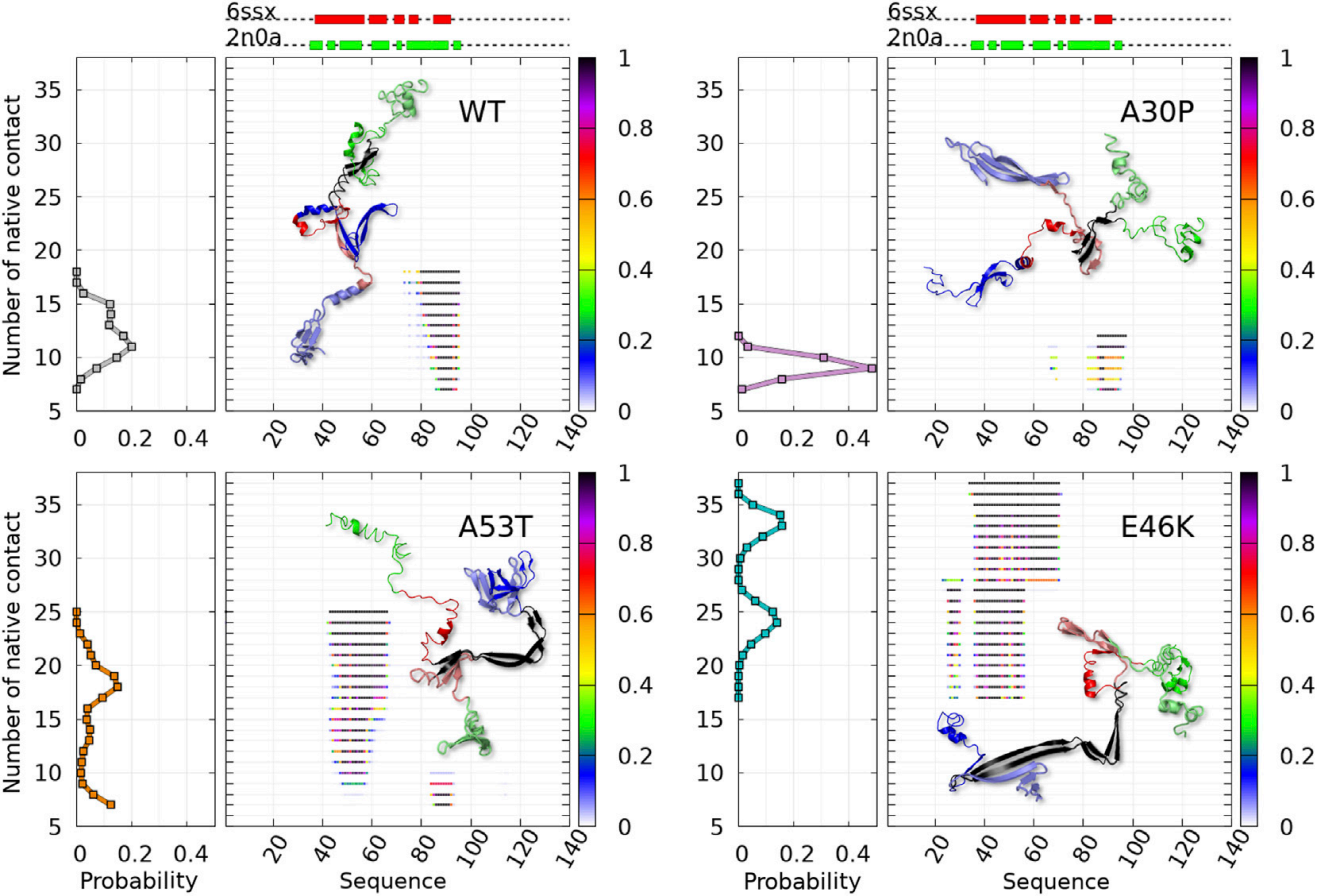
Analysis of the mean contact probability along the amino-acid sequence for the sub-ensemble of Dfncs found in MD trajectories for the WT and variants. For each protein, the left panel represents the probability density of the number of contacts and the right panel represents its distribution along the sequence for each point of the distribution, with a color code giving the probability at each residue according to the color bar on the right. 3D structure representatives of the maximum number of native fibril-type contacts are shown with the following color: N-terminal (blue), NAC (red), C-terminal (green), and native contact region (black).

Figure 8 shows that regions of high propensity of Nfcs in A53T and E46K are significantly different from those of the WT and A30P. This can be more clearly seen for the Nfcs in Figure 9. Figure 9 shows the different lengths of the Nfc regions and their probabilities along the sequence. A30P has the shortest consecutive segments of Nfcs, with the longest between residues 86 and 97 (NAC and C-terminal region), with the largest probability of Nfcs at 87–88 (NAC). The maximum number of consecutive Nfcs for the WT is 18 between residues 78 and 95 (NAC). As already mentioned, the formation of Nfcs in A53T and E46K occurs differently compared to the WT and A30P: they are mainly in the N-terminal region. These two variants have the largest segments of consecutive Nfcs on the time scale of the simulation: 25 between residues 43 and 66 for A53T and 37 between residues 34 and 70 for E46K. The structures of the proteins with the largest number of Nfcs are represented in the insets of Figure 9, and they can be considered the most probable nucleation structures of Dfncs on the time scale of the present simulations (millisecond). As mentioned earlier, the number of Dfncs structures is the largest for A30P and E46K. One may deduce that the nucleation is easier for these two structures. On the other hand, if we consider the maximum number of Nfcs possibly formed on the time scale of the simulations as a criterion in the difficulty of the growth of Nfcs from a nucleation center, we found by increasing order of difficulty: A30P, WT, A53T, and E46K.

As shown in Figure 9, some Dfncs of A53T and E46K have segments that do not start before residue 39 and do not expand above residue 58. It is worth noting that residue 58 is the location of the maximum of probability to form a helix and residue 39 has a high propensity to form intra-molecular *β*-sheets in both disordered dimers (Figure 6) and the monomers (Guzzo et al., 2021).

### 3.4 Selected Local and Global Properties Related to Experimental Techniques

In dimers, the residues may form both intra-molecular and intermolecular parallel and anti-parallel *β*-sheets that can be distinguished in infrared and Raman spectroscopies using amide bands. Table 2 shows a significant variation in the amount of parallel *β*-sheets between the monomers and the dimers. The formation of disordered dimers and Dfncs is clearly characterized by an increase in the proportion of residues in parallel *β*-sheets compared to monomers: by increasing percentage, one finds monomer, disordered dimers, and Dfncs. The amount of parallel *β*-sheets in Dfncs is the largest for the WT despite the fact that the length of segments of Nfcs is quite short (Figure 9), which implies that most of the parallel *β*-sheets are in segments of WT monomers that are not aligned as in fibrils. For the proportion of anti-parallel *β*-sheets, we do not observe a clear systematic variation between the monomers and the dimers. For A53T and E46K, the percentage of anti-parallel *β*-sheets is increased from the monomers to disordered dimers and Dfncs. No significant change is found for the WT. The case of A30P is special: the proportion of anti-parallel *β*-sheets is decreased in Dfncs and increased in disordered dimers compared to the monomers. It is worth noting that on the time scale of the simulations, the Dfncs represent about 9–15% of the structures, with the other dimers being disordered.

**TABLE 2:** Percentages of residues in parallel and anti-parallel intra- and intermolecular *β*-sheets computed with CUTABI for the ensemble of dimers, the sub-ensemble of Dfncs, and isolated monomers.

	Dimer	Dimer	Dfncs	Dfncs	Monomer	Monomer
**Protein**	**Parallel**	**Anti-parallel**	**Parallel**	**Anti-parallel**	**Parallel**	**Anti-parallel**
WT	16.5	27.9	29.1	28.7	10.6	28.4
A30P	18.2	35.1	21.0	27.0	10.9	31.1
A53T	17.1	29.4	22.8	30.8	11.0	22.4
E46K	18.9	31.8	23.1	30.0	10.3	25.8

**FIGURE 10:**
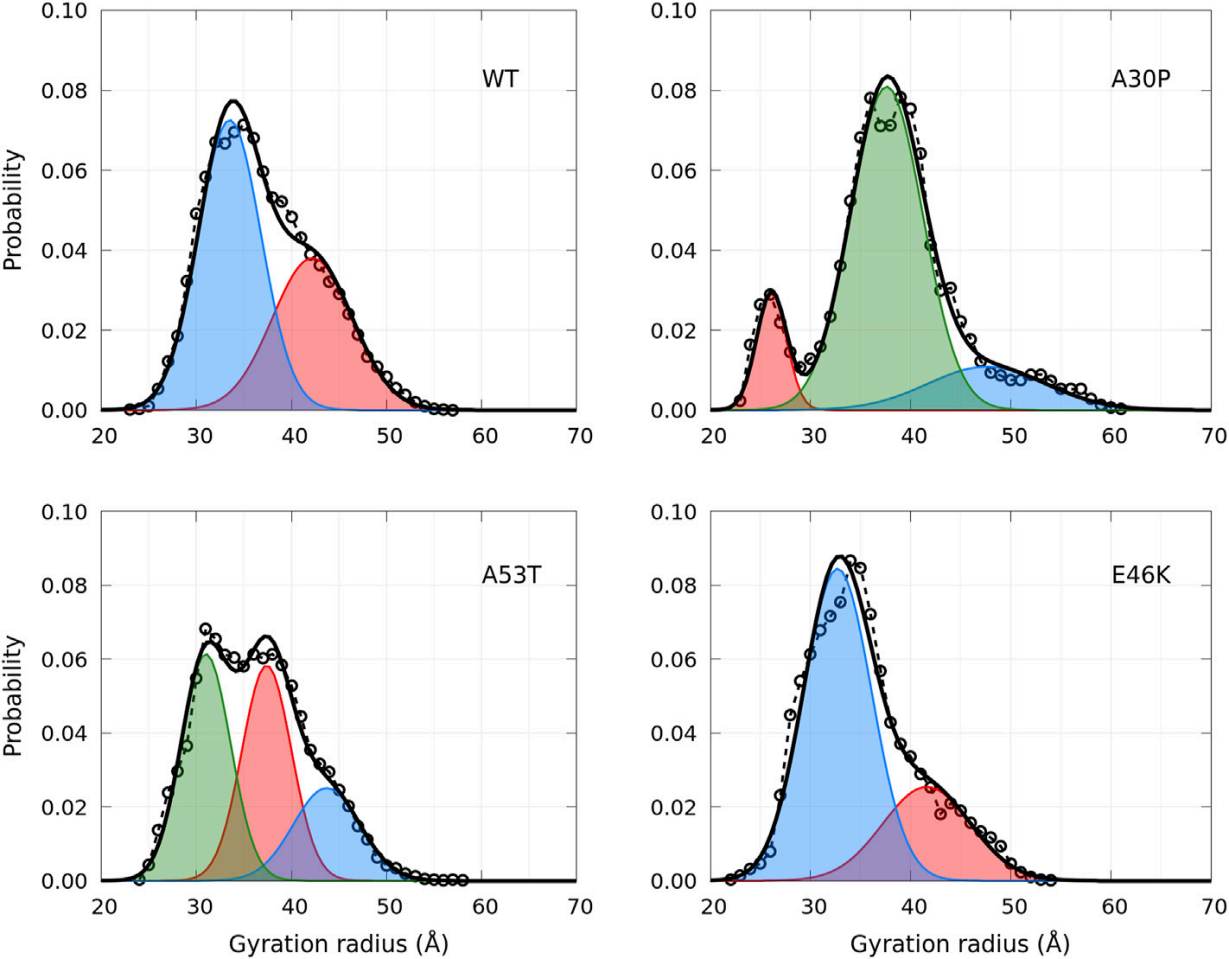
Probability density functions of the gyration radius for the WT and mutants computed from MD simulations (circle symbols and dashed lines). Each distribution is represented by a set of Gaussians (colored areas), the number and the parameters of which were found by applying the GMM algorithm. The sum of Gaussians is represented by a full black line.

A global parameter that can be measured by SAXS is the gyration radius. The probability density functions of the gyration radius of the ensemble of dimers are presented in Figure 10. Each function can be represented by the sum of several subpopulations described by Gaussian functions. The Gaussian parameters, given in Table 3, were computed with the Gaussian mixture model (GMM) algorithm (Reynolds et al., 2009). However, the GMM clustering is misleading as the subpopulations cover large areas of the free-energy landscape maps of the contacts (*n*_*inter*_, *n*_*intra*_) (Figure 1) and of the secondary structures (*α*, *β*) (Figure 4), as shown in Supplementary Figure S1 and Supplementary Figure S2, respectively. Finally, the average gyration radius (Table 3) is the smallest for E46K and the largest for A30P.

**TABLE 3:** Clustering of the gyration radius probability density using the GMM algorithm. The values in brackets are the corresponding % of the ensemble of the conformations.

Protein	Cluster 1	Cluster 2	Cluster 3	Average value
WT	33.5 Å (60%)	42.2 Å (40%)	-	37.0 Å (100%)
A30P	37.6 Å (73%)	47.6 Å (16%)	26.1 Å (11%)	37.9 Å (100%)
A53T	31.1 Å (41%)	37.6 Å (38%)	43.8 Å (21%)	36.2 Å (100%)
E46K	32.7 Å (72%)	41.7 Å (28%)	-	35.2 Å (100%)

**FIGURE 11:**
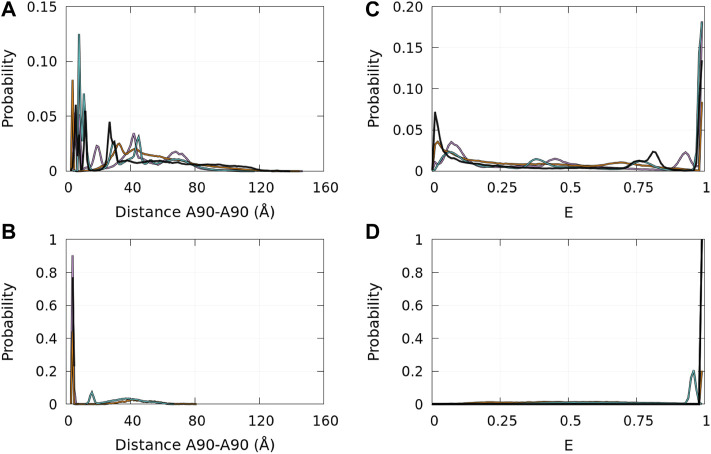
Probability density functions of the distance between the *C* ^*α*^ atoms of residues A90 in the ensembles of disordered dimers [panel **(A)**] and of Dfncs [panel **(B)**] and the corresponding probability distribution functions of the approximated FRET efficiency E (see text) for the ensembles of disordered dimers [panel **(C)**] and of Dfncs [panel **(D)]**. Color code: WT—black, A30P—purple, E46K—turquoise, and A53T—orange.

Single-molecule FRET allows us to extract information on local properties. In Cremades et al. (2012), Horrocks et al. (2015), and Tosatto et al. (2015), the fluorophores (Alexa Fluor 488 and Alexa Fluor 647) were covalently linked at position 90 of *α*-syn mutants A90C. The FRET efficiencies depend on the distance between the fluorophores. Therefore, the FRET experimental procedure observed the aggregation of *α*-syn using a specific local property that is directly correlated with the distance between residues 90 of interacting monomers. It is, therefore, interesting to compute the probability density functions of the distances between the *C* ^*α*^ of residues A90 in disordered dimers and in Dfncs. These functions are presented in Figure 11 (panels A and B) for the WT and mutants. In disordered dimers, we observe a heterogeneity of these distances with a group of peaks below 15 Å and other peaks between 20 Å and 80 Å (panel A). In the subpopulation of Dfncs, there is a drastic change in the distance probability distribution (panel B). All proteins, except E46K, have a peak around 3.8 Å. This can be explained by Figures 8, 9, which show that residue 90 is involved in Nfcs for all proteins, except E46K. All other peaks present in disordered dimers have disappeared in Dfncs, except for E46K, for which we observe a peak at around 15 Å and a large background. Regarding this specific A90–A90 distance, the difference between the probability densities of disordered dimers and Dfncs is spectacular. In addition, this clearly shows that E46K has a different behavior regarding this local parameter.

## 4 Discussion

The first important finding of the present simulations is that the ensemble of *α*-syn conformations (WT and mutants) is strongly heterogeneous, as shown by the computed probability density functions of the gyration radius (Figure 10), the effective free-energy landscape of the contacts (Figure 1), the effective free-energy landscape of the secondary structures (Figure 4), and the probability density function of the A90–A90 distance (Figure 11). The second significant finding is that the ensemble of heterogeneous dimer conformations is divided in a majority of disordered dimers and a complementary minority of Dfncs (Figure 9). The third important finding is that a single amino-acid substitution has a huge effect on the contact probability between (hydrophobic) residues of *α*-syn, which is not limited to the vicinity of the mutated residue as shown in Figure 6 for disordered dimers and in Figures 8, 9 for Dfncs. Keeping in mind that the effective time scale of the present simulations (millisecond) represents the early lag phase of fibril growth, as it is three to four orders of magnitude smaller than the lag phase of fibrils observed experimentally, we will, however, attempt next to make qualitative comparisons between these three significant results and different experimental findings.

Unfortunately, there are no SAXS or SANS measurements of *α*-syn dimers to compare directly with Figure 10 to the best of our knowledge. It is worth noting that the distribution of the gyration radius of *α*-syn monomers is single peak, except for the sub-ensemble of A30P and E46K monomers without an alpha-helix (B monomer state), for which a small peak at *R*_*g*_ = 18 Å was found (Guzzo et al., 2021). For recombinant monomeric *α*-syn, the average gyration radius, extrapolated at infinite dilution, is 27.2 ± 0.44 Å (Araki et al., 2016), comparable to the value calculated previously in our MD simulations (24.7Å) (Guzzo et al., 2021). The experimental value of the gyration radius of a monomer varies among the experiments: for example, in Tris-buffer, it is increased to 42.7Å (Araki et al., 2016). We hypothesize that the presence of dimers in a monomeric solution may give an apparent larger gyration radius of the monomers because the dimer conformational ensembles have an average gyration radius of about 10Å larger than one of the monomer ensembles (Table 2).

The heterogeneity of the size distribution (Figure 10) and of the contacts (Figure 1) of *α*-syn dimers can be related to the rupture-force AFM experiments on WT, A30P, A53T, and E46K *α*-syn dimers in solution (Krasnoslobodtsev et al., 2013). These experiments were performed at low pH to increase the aggregation propensity of *α*-syn and with the additional mutation A40C needed to hang one of the monomers on the AFM tip and the other on the surface. Contour lengths of the dimers were extracted from force–distance curves. They were interpreted as total lengths of stretchable parts of *α*-syn molecules that are not involved in dimeric interaction and, thus, as the sum of the total length from the C-terminal anchor point to the first residue of a dimerization region in each monomer. The data show a multi-peak distribution of contour lengths (Krasnoslobodtsev et al., 2013), which might reflect the multiple minima observed in Figure 1, as the contour length of a dimer conformation is related to the number of intra-molecular and intermolecular contacts. Moreover, the distribution of contour lengths is different for each *α*-syn variant in the AFM experiments, as in Figure 1 for the contacts. We hypothesize that the number of single and multiple rupture force events might be correlated with the propensity of the dimers to form Nfcs. A larger number of multiple rupture force events for A53T and E46K compared to the WT and A30P were interpreted as multiple interaction segments for the former (Krasnoslobodtsev et al., 2013). This might be supported by Figure 8 (black and red curves) as the WT and A30P form Nfcs and intermolecular *β*-sheets in shorter localized segments than A53T and E46K. Moreover, contour lengths can be estimated for residues not involved in Nfcs shown in Figure 8 by assuming a *C* ^*α*^ − *C* ^*α*^ virtual bond distance of 3.8 Å and evaluating the contour length as twice (for the two monomers) the distance starting from the C-terminal to the first residue forming a Nfc (Krasnoslobodtsev et al., 2013). For WT Dfncs, we found a contour length of 357 Å (residues 140 to 94). For A30P Dfncs, the distance is similar. For A53T Dfncs, two contour lengths are estimated: 380 Å (residues 140 to 91) and 585 Å (residues 140 to 64). This last value is the same for E46K Dfncs. These values are on the same order of magnitude than the most probable contour lengths in the experiment (340 Å and 440 Å) (Krasnoslobodtsev et al., 2013). The comparison, although qualitative, indicates that the heterogeneity of *α*-syn dimers and the influence of a single amino-acid substitution on their structural properties, found in MD, agree qualitatively with the AFM force–distance data.

The heterogeneity of the (*α*, *β*) map could be tested in the early lag phase of fibril formation by using single-molecule Raman spectroscopy (Dai et al., 2021), as *α*-helices and *β*-sheets have been well described by Raman fingerprints. Such data are not available so far. In the present simulations, the ensemble of heterogeneous dimer conformations is divided in a majority of disordered dimers and a complementary minority of Dfncs for both the WT and mutants. Dfncs could be identified by spectroscopy. Indeed, the percentage of parallel intermolecular *β*-sheets is characteristic of Dfncs as it is significantly larger than the one in monomers and in disordered dimers (Table 2). This result agrees with FTIR spectroscopy measurements indicating that WT *α*-syn fibrils have a majority of parallel *β*-sheets, contrary to oligomers which show a majority of anti-parallel *β*-sheets (Chen et al., 2015). The total amount of *β*-sheets estimated from FTIR (Chen et al., 2015) is 35 ± 5% for oligomers of 10–40 molecules and 65 ± 10% for fibrils. In spite of the smaller size of dimers and the shorter time-scale of the present simulations, these values compare qualitatively with the sum of parallel and anti-parallel *β*-sheets in WT disordered dimers (44.4%) and in Dfncs (57,8%), respectively (Table 2).

Probably, the best technique to identify Dfncs from disordered dimers would be single-molecule FRET. We tentatively compared the results of Figure 11 with FRET studies of *α*-syn oligomerization (Cremades et al., 2017; Cremades et al., 2012; Tosatto et al., 2015; Horrocks et al., 2015) in the early lag phase of fibril formation. A quantitative prediction of the FRET efficiencies from *α*-syn conformations would require to simulate the protein labeled with the fluorescent molecules and to model the FRET mechanism (Hoefling et al., 2011). Such an approach is difficult to apply for a large and complicated protein like *α*-syn. In addition, to predict protein structure modifications introduced by the cysteine mutations and the fluorescent organic molecules, it is necessary to build and test a new coarse-grained force field for the fluorescent molecules covalently linked to *α*-syn. This task is extremely demanding in computer time and is well beyond the scope of the present work. For these reasons, we chose to compute an approximate efficiency *E* from the distance distributions between the *C* ^*α*^ atoms shown in Figure 11 (panels A and B). From these distances, we extracted an approximated FRET efficiency *E* as $E=1/\left[1+{\left(d/R_{0}\right)}^{6}\right]$ , where *d* is the distance between the *C* ^*α*^ atoms increased by 20Å and *R*_0_ is the Förster distance with *R*_0_ = 60Å for the fluorophores used (dos Remedios and Moens, 1995). Note that a single value of *R*_0_ is a severe approximation that assumes an averaging of the fluorophore orientation. The shift of 20Å takes into account twice the estimated distance between the dye center of a fluorophore and the *C* ^*α*^ atom to which it is linked (Nicolaï et al., 2013).

The probability distribution of *E* is shown in Figure 11 (panels C and D). In disordered dimers (panel C), we observed two regions with peaks of low (<0.15) and of high efficiency (>0.75) . In Dfncs (panel D), peaks are observed only at high efficiency (>0.9) with a small background at all efficiencies. It is tempting to associate the Dfncs with type B dimers. The disordered dimers are both A and B (panel C). It was proposed that the *α*-syn dimers might be a mixture of types A and B, which cannot be resolved experimentally (Horrocks et al., 2015). The time scale simulated here is of course extremely short compared to that in FRET experiments, but it is very interesting to observe that the formation of consecutive Nfcs leads to an increase in the FRET efficiency. It is worth noting that these results are not very sensitive to the exact value of the estimated distance between the dye center of a fluorophore and the *C* ^*α*^ atom. Using a distance of 7.5Å (Supplementary Figure S3) and 15Å (Supplementary Figure S4) instead of 10Å (Figure 11) leads to the same conclusions. Comparison with Figure 9 shows that A90 belongs to all segments of consecutive Nfcs, except for E46K. However, Figure 8 shows that the Dfncs of the E46K variant form other contacts in the A90 region. Thus, E46K behaves differently from the WT, A30P, and A53T in the A90 region, which may be a hint to explain the difficulty in reproducing the probability distribution of the FRET signal in the experiments (Horrocks et al., 2015). If the population of dimers formed in the early lag phase measured by FRET for A30P is taken as 1 for reference, the population of WT and A53T dimers was 0.4 and 0.64, respectively (Horrocks et al., 2015). In the present simulations, the populations of Dfncs using the same reference are 0.59 and 0.75 for the WT and A53T. However, the total populations of dimers out of all the conformations (monomers + dimers) found in the simulations are different. Taking A30P as a reference (=1), we found 1.34 and 1.26 for the WT and A53T, respectively.

The effect of a single amino-acid substitution on the propensity to form contacts (Figure 1) and on the formation of Nfcs (Figures 6, 8, 9) is spectacular. How could these results be compared to various differences observed experimentally between the variants and the WT? The aggregation of A30P in fibrils is slower than the WT (Conway et al., 2000), whereas E46K and A53T aggregate faster than the WT (Conway et al., 1998; Greenbaum et al., 2005). However, the A30P monomer was consumed at a comparable rate or slightly more rapidly than the WT monomer, whereas A53T was consumed even more rapidly (Conway et al., 2000). This might be explained by the early nucleation steps of the fibrils, which is presented by Figure 8. Indeed, E46K and A53T form *larger* regions of Nfcs than the A30P and WT, which might be interpreted as they are “faster” to form pre-fibrils. For A30P, the region of Nfcs is extremely short. However, the sub-ensemble of Dfncs in the dimer ensemble is larger for A30P (14.04%) and E46K (15.73%) in comparison with the WT (8.33%) and A53T (10.65%). On the opposite, the number of dimers found in the MD simulations is the lowest for A30P (23%), followed by an increasing order by A53T (29%), the WT (31%), and E46K (35%). To make these percentages more concrete, it is better to use an example. For 123 monomers in solution, A30P forms 20 disordered dimers, and 3 Dfncs and 77 monomers remain free. For 121 monomers in solution, the WT forms 28 disordered dimers, and 3 Dfncs and 69 monomers remain free. For 129 monomers, A53T forms 26 disordered dimers, and 3 Dfncs and 71 monomers remain free. Finally, for 135 monomers, E46K forms 30 disordered dimers, and 5 to 6 Dfncs and 65 monomers remain free. Thus, all proteins will form approximately the same number of Dfncs, except E46K, but the size of Dfncs on the same time scale is much larger for A53T and E46K (Figure 9).

In order to compare the present results to WT mutagenesis experiments, we list the key residues that play a role in *α*-syn dimerization in the present simulations in Table 4 for the WT and mutants. These residues were selected from the maxima of probabilities of mean contacts (Figure 1) and of the formation of intermolecular *β*-sheets and Nfcs (Figures 6, 8).

**TABLE 4:** Main residues or segments identified by MD as important for the dimerization of *α*-syn from the maxima of propensity for the mean contact, intermolecular *β*-sheets, and Nfcs.

Protein	Mean contact	Intermolecular *β*-sheet (all dimers)	Nfcs in Dfncs
WT	I88	I88	T75, A76, K80, T81,V82, A85, S87-G93
A30P	V70	A90	S87, I88, A90-V95
A53T	L38	L38	T44-N65, S87-T92
E46K	V49	Y39	V26-E28, V37-N65

For the WT, residue I88 is of paramount importance. The segment S87-G93 forms only Nfcs in the sub-ensemble of Dfncs (the black and red curves are superposed in Figure 8). This agrees with the observation that the removal of the segment A85-E94 reduces the *α*-syn polymerization (Waxman et al., 2009). Truncation of V71-V82 prevents the polymerization of fibrils (Waxman et al., 2009). This agrees with the fact that K80-V82 forms Nfcs. We found that the pair T75-A76 has non-negligible probabilities to form Nfcs and other intermolecular *β*-sheets (Figure 8). However, removal of residue A76 or V77 alone has no effect on the polymerization, but the missing pair A76-V77 prevents the polymerization in fibrils (Waxman et al., 2009). The role of A76 cannot be neglected. It is worth noting that Table 4 suggests that mutations or cutting segments in the N-terminus of A53T and E46K could provide information on the key residues promoting the aggregation of these variants.

The role of the N-terminal region in *α*-syn in the dimerization is not to be underestimated as shown by the formation of Nfcs for A53T and E46K and by the high probability to form intra-molecular *β*-sheets for the WT and A30P in this region (Figures 8, 9). In a recent work, two segments in the N-terminal region that regulate the *α*-syn polymerization have been identified: G36-S42 (named P1) and K45-E57 (named P2) (Doherty et al., 2020; Tripathi, 2020). The removal of P1 at pH 7.5 prevents *α*-syn aggregation but not at pH 4.5. The removal of P1 and P2 prevents the aggregation at both pH. The present simulations are calibrated at pH 7 for which the force field was developed. As a single amino-acid substitution has a huge effect on the propensity of aggregation, it is difficult to compare the results of P1 truncated protein with the present results. However, dimerization of A53T and E46K shows that a mutation in this region drastically change the Nfc propensity. Moreover, for the WT, the region G36-S42 is a region with high propensity of intra-molecular *β*-sheets in Dfncs which precedes a region of intermolecular *β*-sheets (43–51) (black curve in Figure 8). It is possible that the formation of intra-molecular *β*-sheets may be coupled to the formation of fibrils. Finally, region P2 overlaps the region of the main helical segment of WT monomers (Guzzo et al., 2021) and dimers (Figures 6, 8). Clearly, the high probability of this helical segment must play a role in the polymerization.

In conclusion, the present MD simulations show that the dimer conformations are largely heterogeneous with both disordered and pre-fibrillar-like dimers that differ between the WT and the variants A30P, A53T, and E46K. Despite the limitations inherent to any MD simulations (accuracy of the force field and the limited time scale), the present findings agree quite well with available experimental data and suggest possible further spectroscopic and mutagenesis experiments.

## Data Availability

The original contributions presented in the study are included in the article/Supplementary Material; further inquiries can be directed to the corresponding author.
