# Supplementary material for: Wild-Type α-Synuclein and Variants Occur in Different Disordered Dimers and Pre-Fibrillar Conformations in Early Stage of Aggregation
Source: Front Mol Biosci. 2022 Jun 28;9:910104. doi: 10.3389/fmolb.2022.910104 (PMC9273784; doi:10.3389/fmolb.2022.910104)
Supplement: Supplementary file 1 [file Presentation1.pdf]

Supplementary Material

SUPPLEMENTARY FIGURES

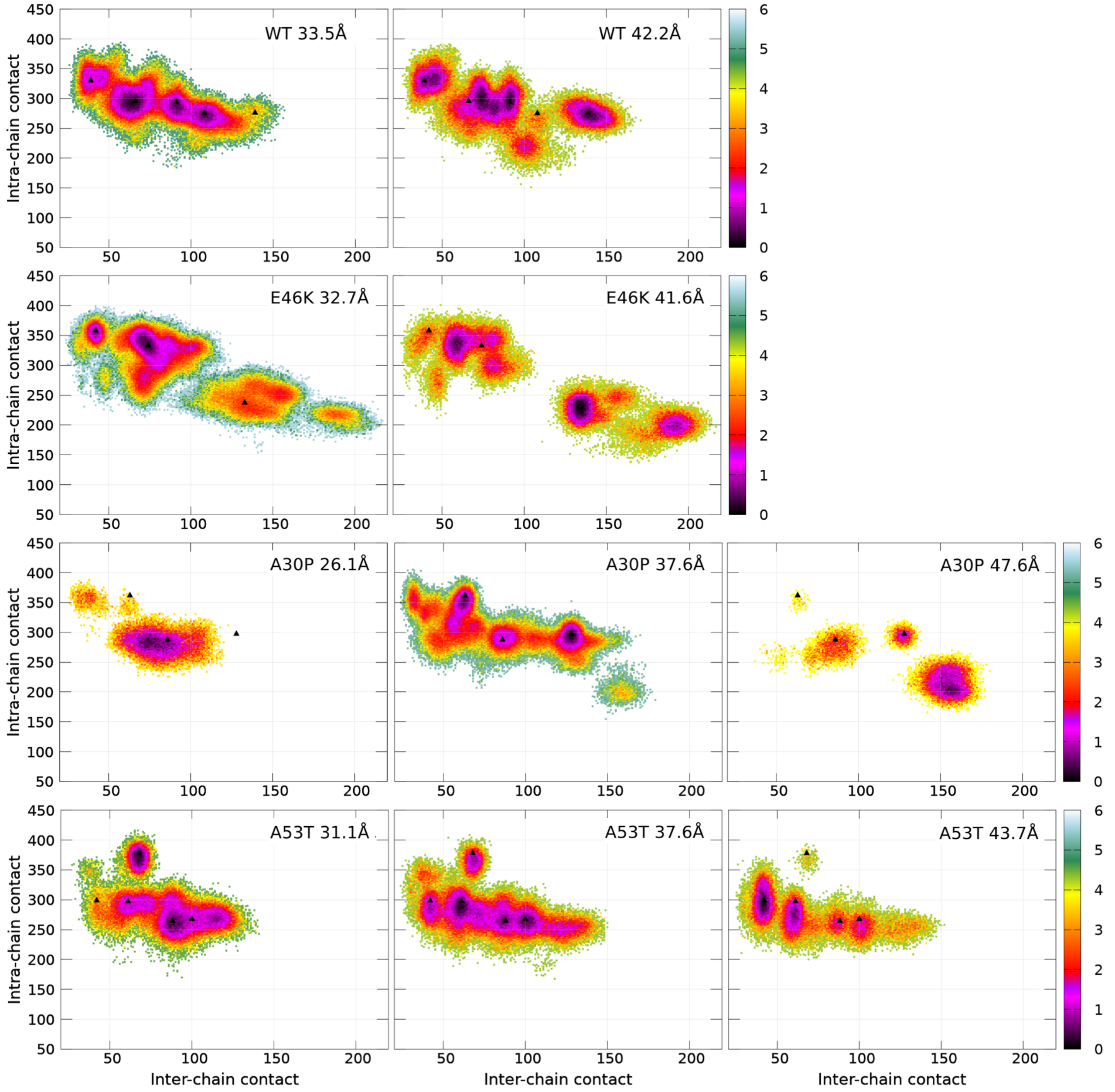

Figure S1. Color maps of -$ln\left[\frac{P(n_{inter},n_{intra})}{P_{max}}\right]$ computed from the 2D probability density function P of the number of inter-chain (n_{inter}) and intra-chain (n_{intra}) contacts between the residues of α-syn for each sub-population of dimers computed by the Gaussian Mixture Model (GMM) algorithm for the probability density of gyration radius of WT and mutants (see Figure 10 of the main text). The numbers given in inset are the positions in Å of the maxima of the Gaussian functions in the GGM clustering (see Table 3 of the main text). In each map, P_{max} is the maximum value of the probability of the map.

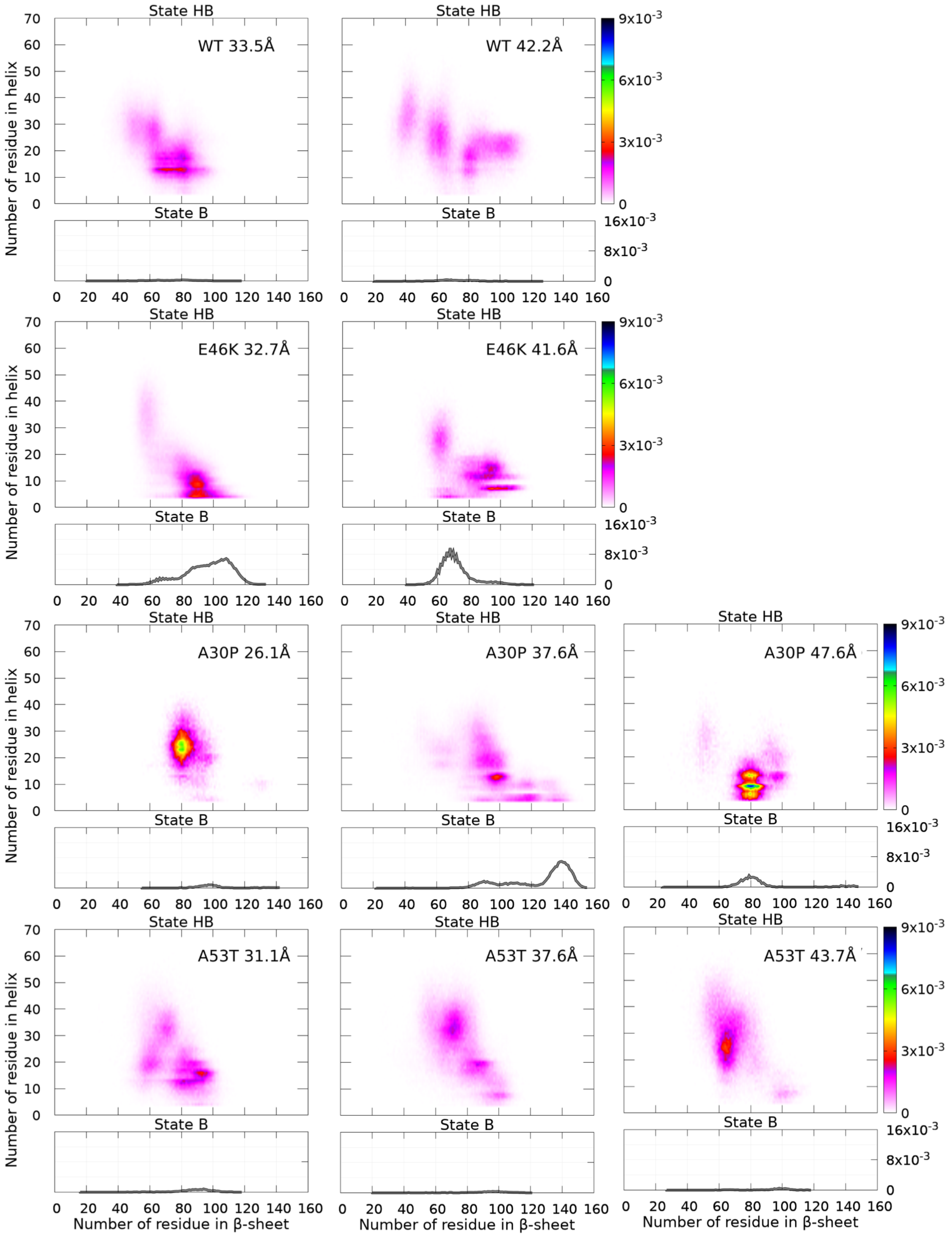

Figure S2. Probability density of the number of residues in α-helix and β-sheet for each sub-population of dimers computed by the Gaussian Mixture Model (GMM) algorithm for the probability density of gyration radius of WT and mutants (see Figure 10 of the main text). The numbers given in inset are the positions in Å of the maxima of the Gaussian functions in the GGM clustering (see Table 3 of the main text). The probability density of the state B (no helix) is represented by a function (gray) (right vertical axis) and the probability density of state HB is represented by a two-dimensional map (right color bar).

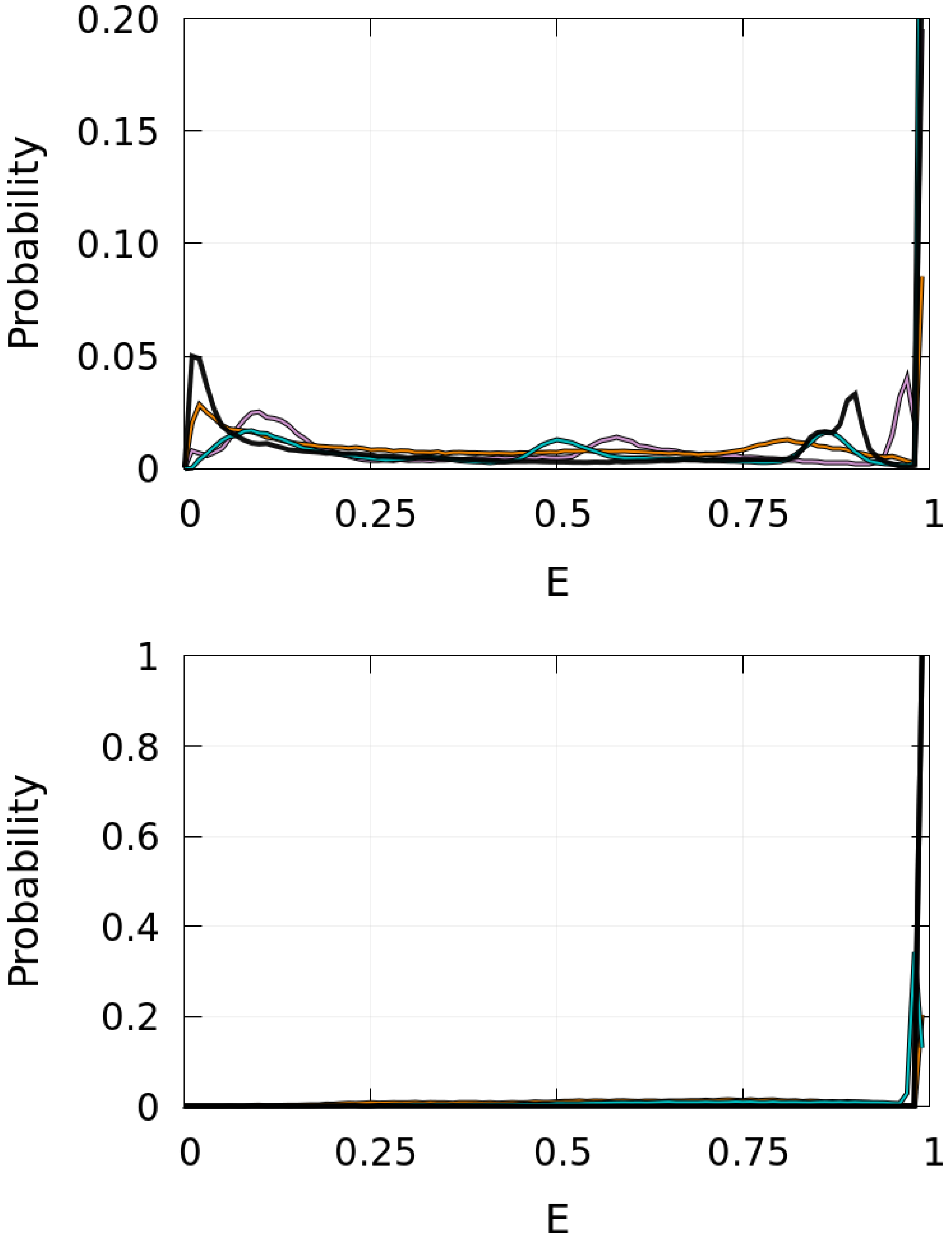

Figure S3. Probability distribution functions of the approximated FRET efficiency E for the ensembles of disordered dimers (upper panel) and of Dfnc (bottom panel), computed by using $E = 1/[1 + (d/R_0)^6]$, where d is the distance between the C^{α} atoms increased by 15Å and R_0 is the Förster distance with $R_0 = 60$Å. Color code is WT (black), A30P (purple), E46K (turquoise) and A53T (orange)

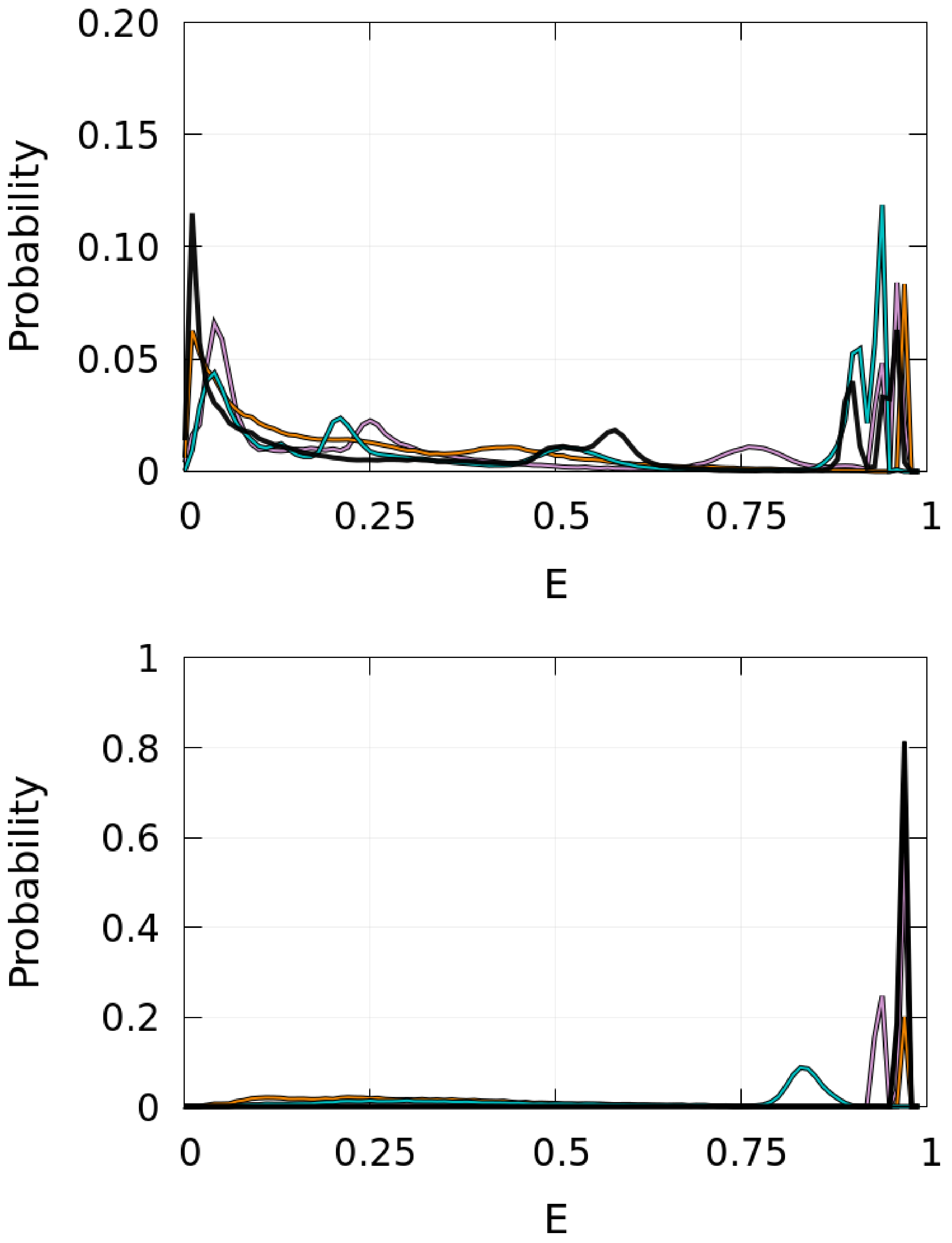

Figure S4. Probability distribution functions of the approximated FRET efficiency E for the ensembles of disordered dimers (upper panel) and of Dfnc (bottom panel), computed by using $E = 1/[1 + (d/R_0)^6]$, where d is the distance between the C^α atoms increased by 30Å and R_0 is the Förster distance with $R_0 = 60$Å. Color code is WT (black), A30P (purple), E46K (turquoise) and A53T (orange)
